# The contribution of phonological knowledge, memory, and language background to reading comprehension in deaf populations

**DOI:** 10.3389/fpsyg.2015.01153

**Published:** 2015-08-25

**Authors:** Elizabeth A. Hirshorn, Matthew W. G. Dye, Peter Hauser, Ted R. Supalla, Daphne Bavelier

**Affiliations:** ^1^Department of Brain and Cognitive Sciences, University of Rochester, RochesterNY, USA; ^2^Learning Research and Development Center, University of Pittsburgh, PittsburghPA, USA; ^3^Department of Liberal Studies, National Technical Institute for the Deaf, Rochester Institute of Technology, RochesterNY, USA; ^4^Department of American Sign Language and Interpreting Education, National Technical Institute for the Deaf, Rochester Institute of Technology, RochesterNY, USA; ^5^Department of Neurology, Georgetown University, WashingtonDC, USA; ^6^Faculté de Psychologie et des Sciences de l’Éducation, Université de GenèveGeneva, Switzerland

**Keywords:** deafness, reading, sign language, orally-trained, short-term memory, phonological awareness, semantic-based memory

## Abstract

While reading is challenging for many deaf individuals, some become proficient readers. Little is known about the component processes that support reading comprehension in these individuals. Speech-based phonological knowledge is one of the strongest predictors of reading comprehension in hearing individuals, yet its role in deaf readers is controversial. This could reflect the highly varied language backgrounds among deaf readers as well as the difficulty of disentangling the relative contribution of phonological versus orthographic knowledge of spoken language, in our case ‘English,’ in this population. Here we assessed the impact of language experience on reading comprehension in deaf readers by recruiting oral deaf individuals, who use spoken English as their primary mode of communication, and deaf native signers of American Sign Language. First, to address the contribution of spoken English phonological knowledge in deaf readers, we present novel tasks that evaluate phonological versus orthographic knowledge. Second, the impact of this knowledge, as well as memory measures that rely differentially on phonological (serial recall) and semantic (free recall) processing, on reading comprehension was evaluated. The best predictor of reading comprehension differed as a function of language experience, with free recall being a better predictor in deaf native signers than in oral deaf. In contrast, the measures of English phonological knowledge, independent of orthographic knowledge, best predicted reading comprehension in oral deaf individuals. These results suggest successful reading strategies differ across deaf readers as a function of their language experience, and highlight a possible alternative route to literacy in deaf native signers.

**Highlights**:

1. Deaf individuals vary in their orthographic and phonological knowledge of English as a function of their language experience.

2. Reading comprehension was best predicted by different factors in oral deaf and deaf native signers.

3. Free recall memory (primacy effect) better predicted reading comprehension in deaf native signers as compared to oral deaf or hearing individuals.

4. Language experience should be taken into account when considering cognitive processes that mediate reading in deaf individuals.

## Introduction

Learning to read, although a rite of passage for most children, remains a significant educational challenge. It is widely known that learning to read is especially difficult for deaf individuals, with the average deaf reader reaching only a fourth grade reading level (Traxler, 2000). For hearing individuals, foundational steps to achieving skilled reading comprehension include becoming aware that words are made of smaller units of speech sounds, a process termed phonological awareness, and then learning to link visual and phonological information to decode print into already known spoken words (Wagner and Torgesen, 1987; Stahl and Murray, 1994; Høien et al., 1995). As they are sounded out, words are then mapped onto their existing semantic representations and knowledge of the syntax and regularities of the language then help the extraction of meaning from text (Wagner and Torgesen, 1987; Cornwall, 1992; Wagner et al., 1994; Hogan et al., 2005). In deaf populations, where there is not necessarily a known spoken language to map the print information onto, becoming a proficient reader poses its own set of challenges. In this study, we ask which component processes mediate reading comprehension in deaf individuals with severe-to-profound hearing loss, and in particular, investigate the impact of phonological knowledge, memory processes and language experience on reading comprehension (Fletcher, 1986; Wagner and Torgesen, 1987; Swanson, 1999; Swanson and Ashbaker, 2000; Scarborough, 2009).

A main determinant of reading in hearing populations remains the mastery of phonological awareness skills, especially those measured at the single word level (Wagner and Torgesen, 1987; Hatcher et al., 1994; Wagner et al., 1994). In young readers, strong phonological representations facilitate word identification skills, which support comprehension (Perfetti and Hart, 2001; Perfetti et al., 2005). Thus, phonological awareness often comes to predict text comprehension (Shankweiler and Liberman, 1989; Hatcher et al., 1994; Wagner et al., 1994), although the role of phonological awareness in reading skill generally decreases with age (Wagner et al., 1997; Parrila et al., 2004). Nevertheless, phonological coding during comprehension can persist into adulthood (Coltheart et al., 1988) and also continues to be linked to reading skill in reading disorders (Bruck, 1992; Elbro et al., 1994; although see Landi, 2010). Accordingly, phonological deficits are often at the source of reading problems (Pennington and Bishop, 2009) and believed to be a main predictor of reading deficits like dyslexia (Snowling, 1998; Gabrieli, 2009). Phonological remediation, or explicit phonological awareness training, often helps to improve reading skill in dyslexic readers, at least when measured at the word level (Eden et al., 2004; Shaywitz et al., 2004).

Despite clear reasons why the link between English phonological knowledge and reading comprehension may be different in deaf individuals with impoverished access to auditory signals, the main focus in most research on reading in the deaf has been based on the established hearing model of reading, which emphasizes the role of phonological processing. However, it is still unclear whether phonological awareness of English is similar in deaf and hearing individuals or used in the same way to facilitate reading (Mayberry et al., 2011; Bélanger et al., 2012a), depending on how it is acquired (LaSasso et al., 2003). An inherent complication is that most standard tasks used to evaluate phonological knowledge in hearing populations require speech production; yet, many deaf individuals are not at ease with vocalizing English. Based on the many strategies for completing a speech-based phonological assessment used in the literature, it remains unclear whether deaf individuals have qualitatively similar phonological awareness of English to that of hearing individuals. It is important to note that deaf individuals have access to other types of phonological knowledge through the use of signed languages. These also have a phonological structure (MacSweeney et al., 2008) that can support higher cognitive processes (Aparicio et al., 2007; MacSweeney et al., 2009; Morford et al., 2011). Given our present focus on what is termed ‘phonological awareness’ in the reading literature, the term ‘phonological’ will refer to phonology of spoken English hereafter. We briefly review below the role of English phonological knowledge, memory processes, and language experience on reading in the deaf.

Several groups have found similarities between deaf and hearing participants in English phonological tasks. Hanson and Fowler (1987) examined deaf signers and found that phonological similarity between English word pairs reduced the reading rate in a speeded lexical decision for both the hearing and the signing deaf individuals, concluding that deaf and hearing participants were using a similar phonetic coding strategy. In another study, Hanson and McGarr (1989) found that signing deaf college students were able to perform a rhyme generation task, but not with the same degree of success as their hearing peers. Sterne and Goswami (2000) argued that deaf readers possess phonological awareness at different levels (i.e., syllable, rhyme, phoneme), although they lagged behind their hearing peers. Nevertheless, a recent meta-analysis by Mayberry et al. (2011) found just as many studies reporting that deaf individuals have phonological awareness as studies that found that they do not.

Large variation in the type of tasks used to assess phonological awareness in the deaf may in part account for this discrepancy (e.g., syllable, phoneme, rhyme; Hanson and Fowler, 1987; Sterne and Goswami, 2000). In addition, some studies have used spoken responses, a standard method used in hearing populations to study phonological awareness (e.g., Luetke-Stahlman and Nielsen, 2003); however, spoken response is potentially problematic, especially for deaf individuals that are not comfortable with vocalizing. Other studies require the manipulation of written words to assess phonological awareness, but doing so inherently involves reading and orthographic processing. To reduce such potential confounds, several studies have adopted picture stimuli and asked for phonological judgments about the English names corresponding to the pictures, which has allowed for a less contaminated measure of English phonological awareness in deaf individuals (Sterne and Goswami, 2000; Dyer et al., 2003; MacSweeney et al., 2008; McQuarrie and Parrila, 2009). These studies suggest some level of phonological awareness in deaf individuals, with some pointing to the importance of orthographic-to-phonological regularities in supporting such knowledge. An important feature of English is that it is an opaque writing system without one-to-one mapping of graphemes to phonemes. There are, however, interesting consistencies in the visual orthography that could lead to alternative visual or orthographic strategies when performing a phonological task (McQuarrie and Parrila, 2009). The extent to which English phonological knowledge in deaf populations is based on orthographic regularities will be examined in Experiment 1. We present novel picture-based tasks, designed to assess English phonological knowledge, with the feature that the orthographic-to-phonological regularity of the test items is systematically manipulated in order to separately assess shallow knowledge (based on orthography) versus deep knowledge (phonological knowledge above and beyond orthography).

While the emphasis on phonological awareness has been productive in motivating best practices in general reading instruction for hearing individuals (Trezek et al., 2010), it may obscure the fact that *comprehension* is the end goal of reading (McCardle et al., 2001). Text comprehension also calls upon more general cognitive processes. Verbal short-term memory has been shown to correlate with reading skill in a wide range of studies (Siegel and Linder, 1984; McDougall et al., 1994; Swanson and Howell, 2001). Serial recall is often used as an assessment of verbal STM, and is known to rely heavily on phonological processes, as exemplified by a rich literature on the phonological loop and its rehearsal mechanism in speakers (Baddeley et al., 1984; Burgess and Hitch, 1999; Melby-Lervåg and Hulme, 2010; Bayliss et al., 2015). Importantly, serial recall and other verbal STM measures have been shown to contribute unique variance in explaining reading skill compared to phonological measures alone, at least in hearing readers (Gathercole et al., 1991; McDougall et al., 1994). A few studies have directly compared short-term memory capacity in deaf and hearing individuals. Studies of either orally trained deaf individuals or deaf native signers suggest a reduced STM span in the deaf, whether tested in English or in American Sign Language (ASL; Conrad, 1972; Bellugi et al., 1975; Boutla et al., 2004; Koo et al., 2008). Evidence suggests that this difference is attributable to language modality rather than sensory deprivation, *per se*, as hearing bilinguals have lower STM span in ASL as compared to when tested in English. The precise source of such span differences remains debated with current hypotheses focusing on lesser reliance on the temporal chunking of units in the visual modality (Hall and Bavelier, 2010; Hirshorn et al., 2012) and on factors that would differentially affect articulatory rehearsal, such as ‘heavier’ phonological units (Geraci et al., 2008; Gozzi et al., 2011) or more “degrees of freedom” in phonological composition in sign languages (Marshall et al., 2011). Despite the evidence for serial span group differences, working memory capacity, which is vital when reading tasks are more demanding, has been shown to be equal for deaf and hearing individuals (Boutla et al., 2002, 2004).

Free recall memory span has also been linked with overall reading skill and comprehension (Dallago and Moely, 1980; Lee, 1986). In contrast to serial recall, free recall is thought to rely more heavily on semantic processing, with greater time on each item allowing for deeper processing (Craik and Lockhart, 1972; Craik and Tulving, 1975; Melby-Lervåg and Hulme, 2010). Accordingly, performance on free recall tests is improved by semantic relatedness (e.g., Hyde and Jenkins, 1973; Bellezza et al., 1976). Furthermore, in contrast to serial recall that heavily relies on rehearsal mechanisms, free recall tasks have longer post-stimulus delays, which are thought to allow for short-term consolidation that aids memory retrieval (Jolicœur and Dell’Acqua, 1998; Bayliss et al., 2015) although this distinction between serial and free recall continues to be debated (Bhatarah et al., 2009). Free recall also has the added benefit of distinguishing between the primacy (recall of initial list items) and recency effects (recall of last list items), such that primacy effects depend to a larger extent on semantic processing, while recency effects reflect a greater contribution of short-term rehearsal and phonological processing similar to what is observed in serial recall tasks (Martin and Saffran, 1997; Martin and Gupta, 2004). This distinction appears relevant when considering predictors of reading. For example, reading-disabled children have been reported to have a decreased primacy effect, but equivalent recency effect, compared to non-disabled readers (Bauer and Emhert, 1984).

Finally, members of deaf communities typically vary greatly in terms of their language background. While around 48% of deaf or hard-of-hearing children use “speech only” as their main mode of communication (Gallaudet Research Institute, 2005), linguistic knowledge within these individuals varies widely. In addition, many early studies examining reading in deaf individuals did not identify whether deaf participants were native users of a signed language, orally trained or users of other forms of communication such as Cued Speech or Signed English. This is likely to be important as having access to a *natural* language from birth has been shown to be a precursor to good reading skill in the deaf (Chamberlain and Mayberry, 2000, 2008; Padden and Ramsey, 2000; Goldin Meadow and Mayberry, 2001). Early exposure to a natural language, be it spoken or signed, is associated with better knowledge of grammar and syntax (Mayberry, 1993), executive functioning (Figueras et al., 2008; Hauser et al., 2008a), and meta-linguistic awareness (Prinz and Strong, 1998); all of these in turn appear to foster better reading comprehension (Chamberlain and Mayberry, 2000; Padden and Ramsey, 2000; Goldin Meadow and Mayberry, 2001). For these reasons, we focus here on two distinct groups of deaf readers with early exposure to a natural language: deaf native signers of ASL, who have very limited spoken English skill, and orally trained deaf, that speak and lip-read English and were exposed to speech-based natural language and educated in mainstream schools with hearing peers, termed hereafter *oral deaf*. In Experiment 2, we seek to determine the relative contribution of English phonological knowledge, English orthographic knowledge, serial recall and free recall to reading comprehension in these two populations of deaf readers.

It should be noted that some additional factors naturally co-vary when sampling from these populations. First, despite our selection of individuals with similar *unaided* levels of hearing loss across these two groups, oral deaf individuals are more likely to use hearing aids or have a cochlear implant (CI), which would increase their *aided* hearing loss and access to auditory information. Second, because deaf native signers use ASL as their primary mode of communication, they are more likely to be (bimodal) bilinguals, and also be reading their second language when faced with English text (Chamberlain and Mayberry, 2008; Morford et al., 2011; Piñar et al., 2011). Recent work on reading in deaf native signers suggest, while they clearly possess knowledge of the phonology of English, they may not make use of that phonological knowledge in the same way as hearing individuals do when reading text for comprehension (Miller and Clark, 2011; Bélanger et al., 2012a,b, 2013). It should also be acknowledged that the relative contribution to the reading process of different language experience (such as use of a signed language) and of reading a first versus a second language remains understudied.

In sum, Experiment 1 presents newly developed ‘deaf-friendly’ measures of English phonology that manipulate whether a ‘phonological’ task can be solved with an orthographic strategy or not. In doing so, it allows us to separately assess orthographically based phonological knowledge from non-transparent, deep phonological knowledge of English in deaf readers. Experiment 2 then turns to the determinants of reading in our two groups of deaf adults with different language backgrounds by considering the relative contribution of various types of English phonological knowledge that are based upon the phoneme level (both shallow and deep) and larger phonological units (syllable and speechreading measures), linguistic short-term memory (serial recall span) and semantic-based memory (free recall span). Together, this battery is designed to distinguish between various levels of English phonological knowledge and more general cognitive measures as predictors of reading comprehension in our two groups of deaf adults. Based on the existing literature, we predicted weaker deep phonological knowledge in deaf native signers than in the oral deaf. Moreover, we hypothesized that reading comprehension may show a greater reliance on memory processes, especially semantic-based, in deaf native signers, whereas deep phonological knowledge would be the primary predictor of reading skills in the oral deaf.

## Experiment 1

The goal of Experiment 1 was to determine the extent and type of English phonological knowledge in two groups of deaf readers. More specifically, we tested the extent to which the two deaf groups utilized visual orthographic knowledge to complete phonological tasks. Two new tests of English phonological knowledge were designed for use with our profoundly deaf participants. An important design feature that was we did not want to require vocal responses or use text-based materials to measure phonological knowledge, making commonly employed tasks like non-word naming inappropriate. Instead our tests require button-press responses and use nameable black and white pictures to provide a cleaner measure of phonological knowledge – there is no explicit phonological representation in the picture itself, unlike for written words. Critically, the transparency of the orthographic-to-phonological mapping was systematically manipulated in order to assess how much a purely orthographic strategy was being used to perform a phonological task. More specifically, the transparency of orthographic-to-phonological mapping was explicitly manipulated such that orthographic information, if used, could either help task performance (shallow task) or be uninformative or counter-productive (deep task). This manipulation was deployed in two separate tasks. The first task required participants to indicate which of three items sounded different from the other two, with the difference being sound-based and located either at the first consonant or vowel. The second task mirrored a phonemic manipulation task often used in the reading literature. Participants were asked to extract the first sound and the last sound of the names corresponding to two pictures, and then combine those to make a new name. We expected to see differences between the deaf groups in the extent to which they utilized an orthographic strategy, with deaf native signers using those strategies more than the oral deaf. We note that a group of hearing participants was also evaluated on these tasks to verify that our stimuli properly assess orthographic and phonological knowledge. Their data are reported in the supplementary information and confirm a gradient from shallow to deep phonology with our materials.

### Methods

#### Participants

The study included 26 profoundly deaf native signers of American Sign Language [*M*_age_ = 22 (18–32); 17 female*; M_unaided PTA loss in better ear_* = 94 dB, 73–110 dB; Note PTA means Pure Tone Average] and 21 oral deaf (*M*_age_ = 21 (18–24); 16 female; *M_unaided PTA loss in better ear_* = 90 dB, 63–120 dB). All participants were recruited from the Rochester Institute of Technology (RIT) or the National Technical Institute for the Deaf (NTID).

Inclusion criteria for all participants were: (i) unaided hearing loss of 75 dB or greater in the better ear^1^, (ii) onset of deafness before 2 years of age^2^, and (iii) being right handed. We were unable to acquire the unaided dB loss level for four oral deaf participants and five of the deaf native signing participants. Based upon deaf participants for whom audiological data was available, the two deaf groups had equivalent levels of unaided dB loss (see **Table 1**). Hearing loss levels were obtained from self-reports as well as consented and IRB-approved access to RIT/NTID records. All participants were treated in accordance with the University of Rochester’s Research Subjects Review Board guidelines and were paid for their participation in the study. No participants reported having any learning disorder.

**Table 1:** Demographic and language backgrounds of participants (mean scores with ranges or SD).

Measure	Oral Deaf	Deaf native signers	*t*	*df*	*p*
Age	21 (18–24)	22 (18–32)			
dB loss	87 (16)	90 (10)	0.74	36	0.47
TONI standardized score	98.3 (10.6)	99.4 (10.6)	0.34	38	0.73
Native language fluency					
English	31% (18%)	NA			
ASL	5% (9%)	63% (14%)	14.48	45	<0.001

Additional inclusion criteria for deaf native signers included: being born to deaf parents and exposed to ASL from infancy; and having limited spoken English skill, as measured by the TOAL-2 (see below). All deaf native signers reported having used hearing aids at some point in their lives, but only six continued to use hearing aids regularly and three reported using them only occasionally. Twenty of the deaf native signers attended a school for the deaf during at least one phase of their education before college, and six attended a mainstream school throughout.

In contrast, additional inclusion criteria for oral deaf subjects included: being born to hearing parents; being educated in mainstream schools that adopted oral-aural approaches promoting spoken language ability; minimal or absent ASL skills with no exposure to ASL until college years (average of 2.5 years in college; range = 0.5–6 years); using oral communication as the primary mode of communication; and relying on lip-reading to comprehend spoken English. Most of these students received individual speech therapy on a regular basis upon entering the school system and continued to receive speech training and gained skill in speechreading as a part of all of their academic courses. Four of the oral deaf participants had received CI with an age of implantation of 2.5, 5, 17, and 19 years. Of the 17 oral deaf participants without CIs, all wore hearing aids except two. If participants wore CIs or hearing aids, they were instructed to use them as they normally would during all tasks. Six attended a preschool for deaf children, but all attended mainstream schools during their elementary, middle, and high school years. Fourteen participants reported not using ASL at all, while seven reported having some ASL experience starting in college.

In order to verify participants’ native language proficiency and to confirm that the groups had distinct and separable language skills, we administered ASL and spoken English proficiency tests that probed both comprehension and production. The American Sign Language Sentence Reproduction Test (ASL-SRT) was used as a test of ASL proficiency (Hauser et al., 2008b; Supalla et al., 2014), and the Test of Adolescent Language Speaking Grammar Subtest (TOAL-2; Hammill, 1987) was used as a test of English proficiency. In both tests, subjects saw/heard sentences of increasing complexity and length and were instructed to repeat back exactly what they saw/heard. Thus, both tests involved both a comprehension and a production component. Only sentences recalled verbatim were counted as correct. Deaf native ASL signers scored the ASL proficiency test (for native signers and oral deaf subjects) and hearing native English speakers scored the English test for oral deaf subjects. The percent accuracy (number of sentences repeated verbatim divided by the total) on each proficiency test was compared between groups (see **Table 1** for mean values). For the spoken English proficiency test, deaf native signers were instructed to respond in ASL if they were not comfortable producing overt speech. Nevertheless, native signers were at floor and therefore a statistical test was not needed. **Table 1** shows performance of the two deaf groups on these two sentence repetition tests. For the ASL-SRT, the native signers were more accurate than oral deaf participants. Overall, the language proficiency results confirmed successful enrollment of two groups of deaf participants with distinct language backgrounds: one group is significantly more skilled in spoken English, and the other more skilled in ASL.

Finally, participants completed the TONI-3 (Brown, 2003) to confirm that the two groups did not have significantly different levels of non-verbal IQ in order to control for the impact of general cognitive factors in reading comprehension. Participants viewed arrays of visual patterns of increasing complexity, with one missing component in each array. They were required to identify the missing component by selecting from 4 or 6 options. Due to a communication error early during data collection, some participants were not given the TONI-3 and thus data are missing for one oral deaf, and six deaf native signers. As can be seen in **Table 1**, TONI-3 scores across groups were not significantly different.

#### Design and Procedure

The tasks required phonological judgments to be made on the basis of black and white drawings of objects. It was therefore important to ensure that participants knew the desired English names to be associated with the pictures we used. All participants initially named the pictures by typing their corresponding English name into the computer. There was feedback to make sure they had assigned the correct name and spelling. If a picture was misnamed or misspelled, participants were informed of the mistake and it was presented again at a later time until all pictures had been named and spelled correctly. Instructions were written for oral deaf (and hearing, see Supplemental Information) participants, but the experimenter always reviewed the instructions verbally before the experiment started. An instructional video in ASL was made for signers by a bilingual hearing signer, and gave many examples to ensure the tasks were clear. An ASL/English interpreter skilled in communicating with deaf individuals of varied language background was always present in case clarifications were needed.

##### Phoneme Judgment Task

The Phoneme Judgment Task employed an ‘odd-man-out’ paradigm: three pictures were displayed in a triangle formation on a computer screen, and participants were instructed to select the item with a different sound. Participants responded by pressing ‘H’, ‘B’, or ‘N’ on a QWERTY keyword, corresponding to the ‘odd-man-out’ location on the screen. The odd-man-out could be located either at the first consonant or at the vowel. These two phoneme-type conditions were run blocked with the order of blocks counterbalanced across groups. Words in the first consonant condition could be either one or two-syllables, while the words in the vowel condition were all one-syllable.

**FIGURE 1:**
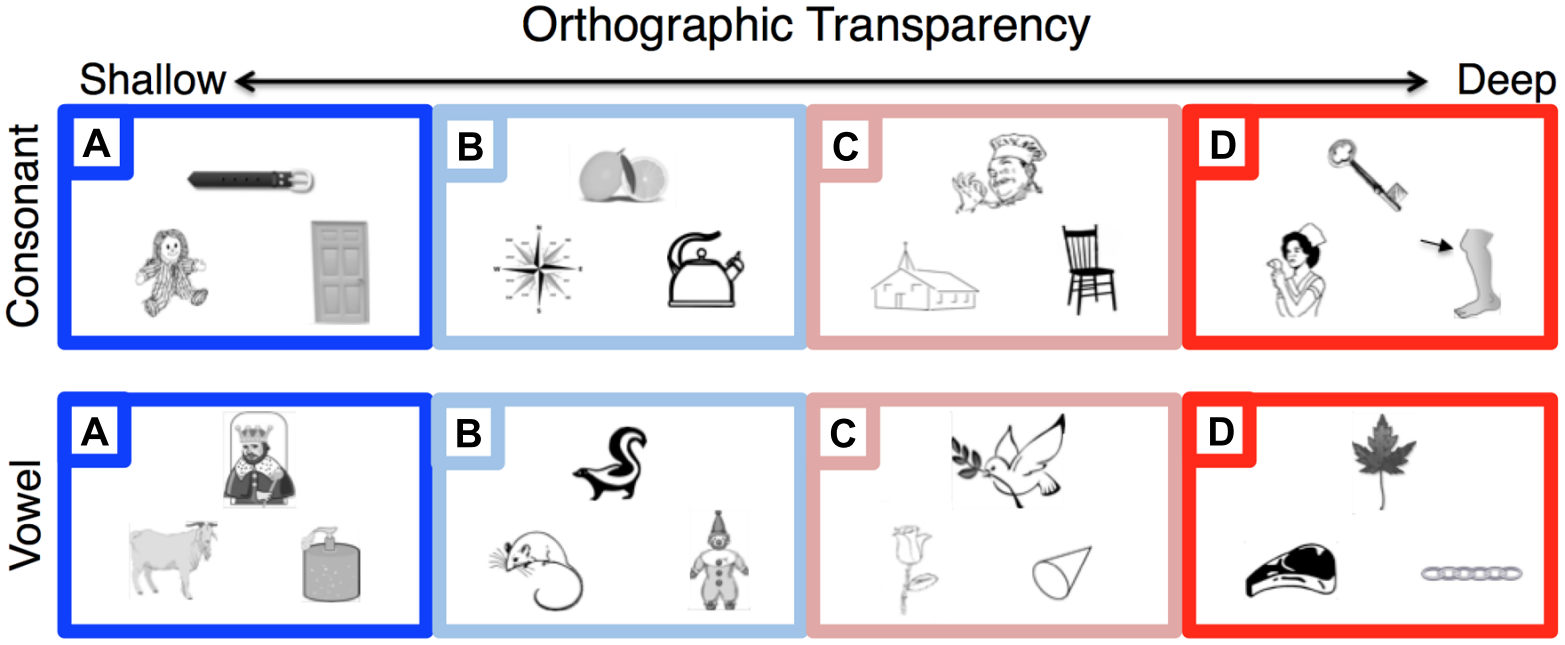
**Example stimuli for Phoneme Judgment Task.** Participants had to pick the ‘odd man out,’ or which of the three pictures corresponded to an English name with a different first consonant sound (top row) or vowel sound (bottom row). For example, belt was the correct answer in the belt/doll/door triplet (top left). The orthographic transparency was manipulated in a graded manner such that orthographic information could help to accurately complete the Shallow (blue) conditions **(A,B)**, but would be uninformative or counter-productive in the Deep (red) conditions **(C,D)**. Shallow **(A)** trials were the most transparent, such that orthography alone could lead to the correct answer (e.g., first consonant: belt/doll/door; vowel: king/goat/soap). Shallow **(B)** trials could also be solved using more advanced orthographic knowledge (e.g., first consonant: lemon/compass/kettle; vowel: skunk, mouse, clown). Deep **(C)** trials did not give any orthographic cues, as all stimuli shared the same orthography of interest (e.g., first consonant: chef/church/chair; vowel: dove/rose/cone). Deep **(D)** trials gave counterproductive information such that using orthographic cues would systematically produce the wrong answer (e.g., first consonant: key/nurse/knee; vowel: leaf/steak/chain). The location of the odd man out was counterbalanced within a participant, but was placed at the top in each example above for clarity.

The complex letter-to-sound mappings of English were exploited in order to determine whether participants were able to go beyond purely orthographic strategies in order to perform accurately. Two conditions were labeled as “shallow” and these were conditions in which a purely orthographic strategy could yield 100% accuracy. In shallow condition A, the similar sounding pair shared the same orthography whereas the odd-man-out had a different orthography (e.g., **b**elt/dog/door for the first-sound task; k**i**ng/goat/soap for the vowel task). In shallow condition B, 100% accuracy using an orthographic strategy would depend upon flexible letter-to-sound knowledge, such as being aware that ‘k’ and ‘c’ can both be mapped to the same sound in English (e.g., **l**emon/kettle/compass for the first-sound task; sk**u**nk/mouse/clown for the vowel task). Another two conditions were labeled as “deep” and were constructed such that accuracy would be poor if an orthographic strategy were employed. In deep condition C, all of the words shared the same letter (e.g., **ch**ef/church/chair for the first-sound task; d**o**ve/rose/cone for the vowel task). This condition therefore requires knowledge of idiosyncratic mappings in English: knowing that ‘c’ can sometimes sound the same as ‘s’ no longer provides a cue to the correct answer. Finally, deep condition D was constructed such that an orthographic strategy would routinely lead to the incorrect answer. In this condition, the odd-man-out shared orthography with one of the two similar-sounding items (e.g., **k**ey/knee/nurse for the first-sound task; l**ea**f/steak/chain for the vowel task). Examples and more details are provided in **Figure 1**. Before each task, instructions were given using two sample trials. The sample trials contained one ‘shallow’ and one ‘deep’ trial to clarify the instructions, but also to demonstrate how they could not always be solved based on orthography alone.

##### Phonemic Manipulation Task (Onset/Rime)

**FIGURE 2:**
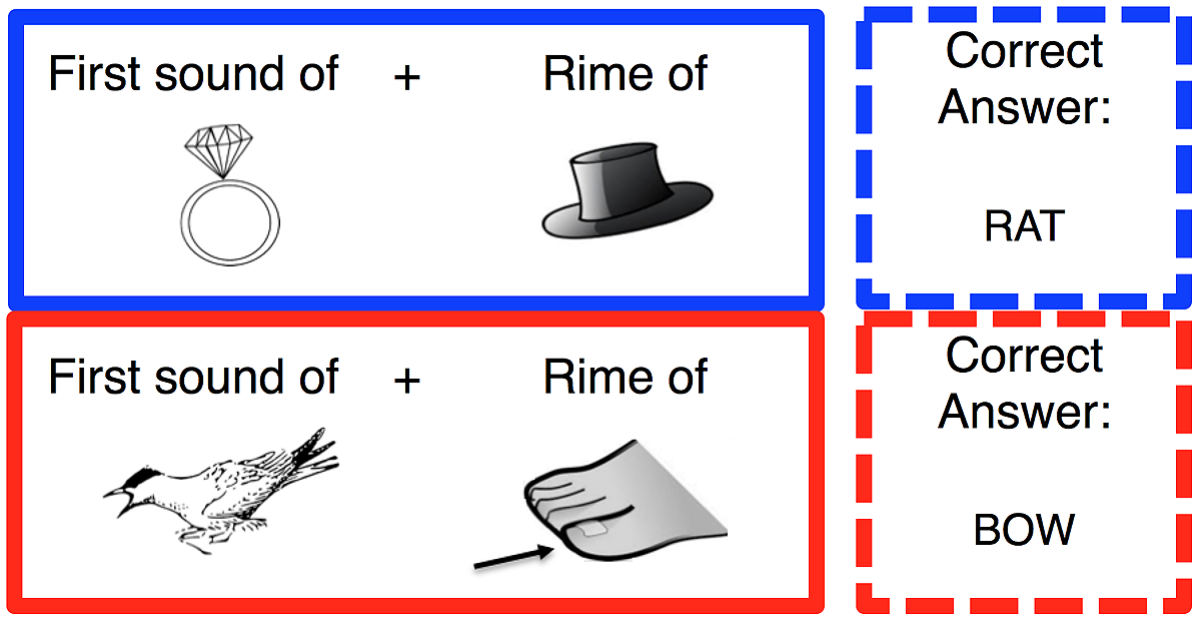
**Example stimuli in the Phonemic Manipulation Task.** Participants were told to take the first sound from the left image and the rime of the right image to make a new word. The shallow trials (blue) were designed so that the task could be completed based on orthography alone (e.g., the first sound of ‘Ring’ + the rime of ‘hAT’ = RAT). The deep (red) trails were designed such that orthography alone could not be used to accurately complete the task (e.g., the first sound of ‘Bird’ + the rime of ‘tOE’ = BOW). The correct answer was not provided to participants as feedback, but is provided in the figure for clarity.

The Phonemic Manipulation Task was to take the onset of a first word (e.g., **R**ing) and the rime of a second word (e.g., h**AT**) to make a new real word, in this case **RAT**. Participants were instructed ahead of time about the difference between the onset (first sound) and the rime of a word, and were given many examples as well as several practice trials. All words used in this test were monosyllabic and, again, only pictures were used as stimuli (see **Figure 2**). Trials differed as to whether they could be completed correctly based on orthography alone, like the example above (called “shallow” trials), or could not (e.g., onset of ‘**B**ird’ plus the rime of ‘t**OE**’ makes a new word ‘**BO*W***’; called “deep” trials). Both shallow and deep trials were administered in the practice session. All subjects responded by typing their answer into the computer.

### Results: Experiment 1

#### Phoneme Judgment Task

**FIGURE 3:**
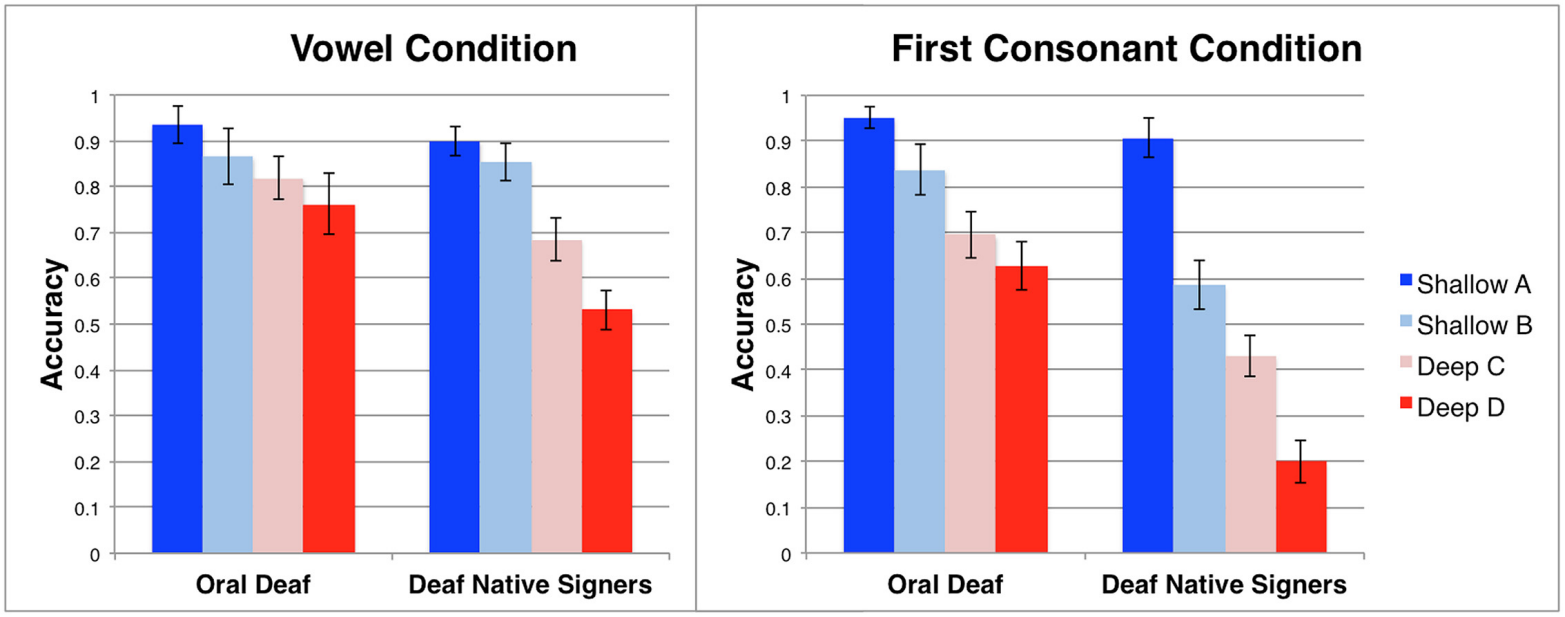
**Performance on Phoneme Judgment Task (Vowels and Consonants) across groups.** The orthographic transparency was manipulated in a graded manner (see **Figure 1**) such that orthographic information could help to accurately complete the Shallow (blue) conditions (A,B), but would be uninformative or counter-productive in the Deep (red) conditions (C,D). Error bars represent SE of the mean.

A 4 × 2 × 2 ANOVA was conducted with *orthographic transparency* (A, B, C, D) and *phoneme type* (consonant, vowel) as repeated measures, and *group* (deaf native signers, oral deaf) as a between subjects factor (see **Figure 3**). The main effect of orthographic transparency, *F*(3,135) = 67.40, η^2^ = 0.60, *p* < 0.001, was significant in the predicted direction: the conditions that could be solved by transparent spelling alone were more accurate than those that required knowledge of the orthographic-to-phonological regularities, with the condition where an orthographic strategy would lead to consistently incorrect responses being the worst. There was a main effect of phoneme type, *F*(1,45) = 22.13, η^2^ = 0.33, *p* < 0.001, such that responses in the vowel condition were more accurate than those in the consonant condition. Lastly, there was a main effect of group, *F*(1,45) = 23.43, η^2^ = 0.34, *p* < 0.001, such that the oral deaf were more accurate than the deaf native signers. All three two-way interactions were significant. The orthographic transparency × group interaction was significant, *F*(3,135) = 8.83, η^2^ = 0.16, *p* < 0.001, such that deaf native signers performance decreased more sharply as orthographic transparency diminishes than that of the oral deaf. The phoneme type × group interaction was significant, *F*(1,45) = 6.00, η^2^ = 0.12, *p* = 0.02, such that the deaf native signers performed relatively worse on the first consonant condition, compared to the vowel condition, than did the oral deaf. Lastly, there was a significant orthographic transparency × phoneme type interaction, *F*(3,135) = 9.24, η^2^ = 0.17, *p* < 0.001, such that the effect of orthographic transparency was more pronounced in the first consonant condition compared to the vowel condition. There was no significant three-way orthographic transparency × phoneme type × group interaction, *F*(3,135) = 2.01, η^2^ = 0.04, *p* = 0.12.

#### Phonemic Manipulation Task

**FIGURE 4:**
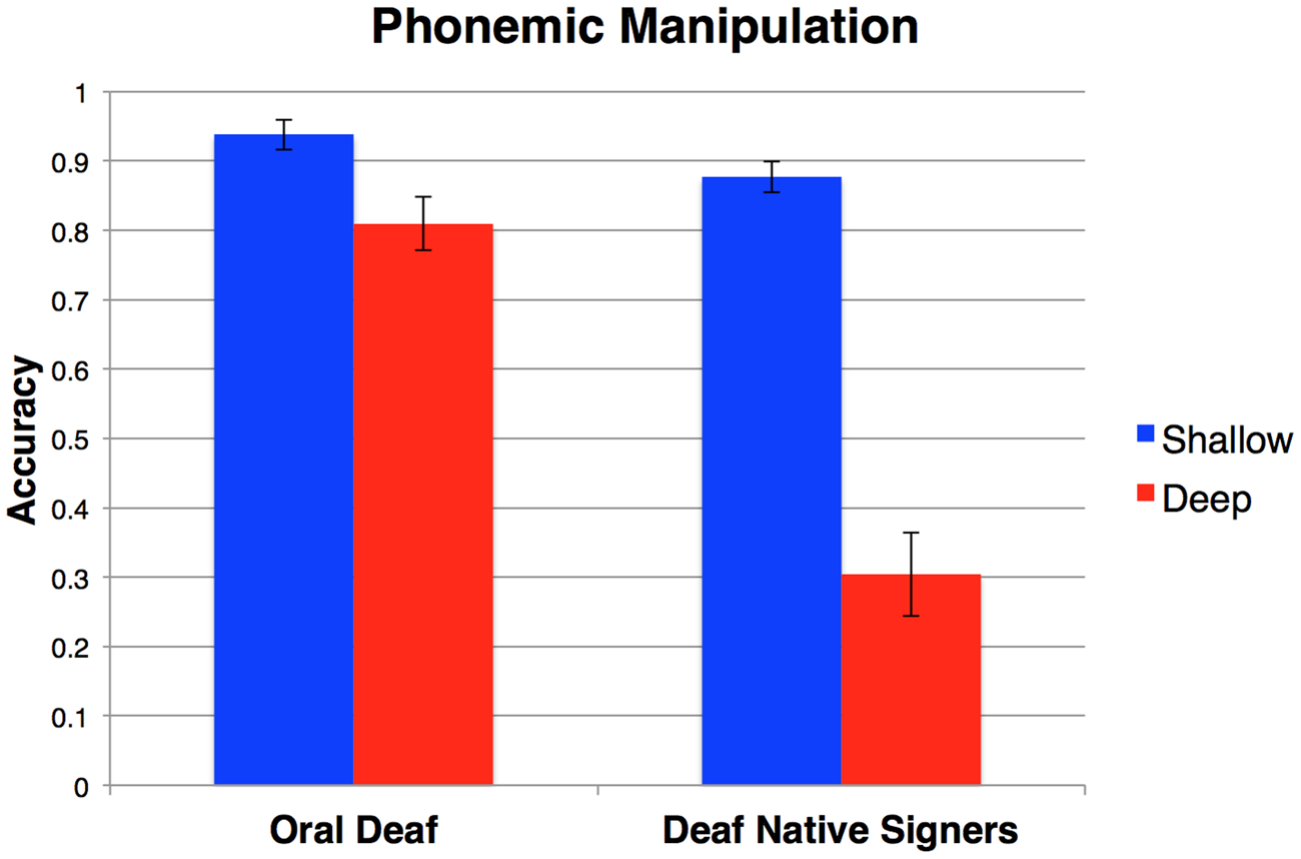
**Accuracy on the Phonemic Manipulation Task across groups.** The shallow condition (blue) could be solved using an orthographic strategy, while the deep (red) condition required phonological knowledge above and beyond orthography. Error bars represent SE of the mean.

Data from the Phonemic Manipulation Task was entered into a 2 × 2 ANOVA with orthographic transparency (shallow, deep) as a repeated measure and group (oral deaf, deaf native signers) as a between subjects factor (see **Figure 4**). There was a significant main effect of orthographic transparency, *F*(1,45) = 96.25, *η^2^* = 0.68, *p* < 0.001, such that participants were less accurate in the deep condition where a transparent orthographic strategy could not be used successfully compared to the shallow condition. There was also a significant main effect of group, *F*(1,45) = 41.86, η^2^ = 0.48, *p* < 0.001, such that the oral deaf had greater accuracy than deaf native signers. Lastly, there was a significant interaction between orthographic transparency and group, *F*(1,45) = 38.63, η^2^ = 0.46, *p* < 0.001, such that deaf native signers performance decreased more sharply from shallow to deep than did the oral deaf performance.

For the separate group of hearing participants run to validate the tasks in Experiment 1, we confirm a significant effect of orthographic transparency in the Phoneme Judgment Task, the Phonemic Manipulation Task and when comparing the Phoneme Composite Scores (see Supplemental Information).

### Experiment 1 Summary

Experiment 1 used two different tasks that systematically manipulated the extent to which orthographic information could be relied upon to access phonemic information. As expected, there was a strong effect of orthographic transparency on accuracy such that responses in shallow conditions were more accurate than in deep conditions. Although both deaf groups were sensitive to orthographic transparency, its impact was more pronounced in deaf native signers. This was the case for both the Phoneme Judgment Task and the Phonemic Manipulation Task. In terms of phoneme types, the vowel condition was easier overall than the consonant condition. Indeed, in the consonant condition of the Phoneme Judgment Task, performance in both deaf groups decreased sharply as orthography became less informative or counter-productive, and this effect was less pronounced in the vowel condition. One may speculate that this may reflect the fact that vowels tend to be more overtly enunciated on the lips (e.g., /e/ and /o/ are clearly differentiated on the lips), whereas many consonant distinctions are impossible to see on the lips (e.g., /ch/ vs. /sh/ or /g/ vs. /k/). Accordingly, greater accessibility through speechreading has been suggested to influence phonological knowledge in deaf populations in previous works (Erber, 1974; Walden et al., 2001).

Overall the main emerging pattern is that both deaf populations have a robust knowledge of orthographic regularities in English; however, deaf native signers show a greater reliance on visual orthographic information than the oral deaf when asked to complete English phonological tasks, at least when tested at the level of individual phonemes.

## Experiment 2

The goal of Experiment 2 was to determine the best predictors of reading comprehension within each group, and compare how they may differ across the two deaf populations. Along with phonological knowledge, the contributions of memory skills that tap either phonological or semantic processing were also assessed in each group. Experiment 2 aims to determine how *useful* these skills may be in the service of reading comprehension in each of these deaf populations and whether group differences may emerge in best predictors. More specifically, we predict that oral deaf, with greater experience with spoken English, will make greater use of speech-based skills than deaf native signers (Lichtenstein, 1998).

A test of English reading comprehension was selected to evaluate reading skill, as many deaf adults, especially native signers, report that it is unnatural for them to read aloud. All participants completed the Peabody Individual Achievement Test-Revised: Reading Comprehension (Markwardt, 1989). This particular test is well tailored to deaf populations as it evaluates reading comprehension at the sentence level via non-verbal responses and has no speech production requirement (Morere, 2012). Participants were the same as in Experiment 1, meaning that the groups’ performance on the TONI-3, a test of non-verbal spatial intelligence (Brown et al., 1997), did not significantly differ.

In addition to reading comprehension, measures known to be linked to reading comprehension skill were collected in order to assess if they differentially predicted reading comprehension across groups. These measures assessed knowledge of English phonology at different levels (Shallow and Deep Phoneme Composite Scores, Syllable Number Judgment, and Speechreading) and also different aspects of memory (serial recall span, primacy in a free recall span task).

### Methods

#### Design and Procedure

##### Reading comprehension

The Peabody Individual Achievement Test-Revised: Reading Comprehension requires participants to read sentences one at a time and decide which of four pictures best matched the sentence just read. As the test progressed, the sentences increased in length, contained a greater number of clauses, and used less frequent vocabulary. Non-matching pictures were foils designed to represent erroneous interpretations that are based on expectations, and not on careful reading of the text. Thus, a reader must completely understand the grammar and vocabulary of the sentence in order to select the correct picture match. Instead of focusing on print-to-sound reading, as many reading tests do, this test focuses on lexical *and* syntactic knowledge of English. This test has been shown to be well suited to deaf populations (for a critique in hearing populations, see Keenan et al., 2006).

##### Phonological measures

Shallow and Deep Phoneme Composite Scores were derived from Experiment 1. In addition, performance on two other phonological tasks was collected. These tasks tapped larger units of English phonology, respectivively syllabic structure and sentence-level speechreading ability.

###### Phoneme Composite Scores

Accuracy on the Phoneme Judgment Task and the Phonemic Manipulation Task from Experiment 1 was collapsed across conditions to produce two composite scores. The first reflects performance in transparent conditions and was termed the ***Shallow Phoneme Composite Score.*** It was derived from mean performance on the first two levels in the Phoneme Judgment Task (A, B) and from the shallow condition in the Phonemic Manipulation Task. The second reflects performance when spelling-to-sound correspondence is challenging, either because of the use of subtle featural differences (e.g., chef versus chair) or irregular orthography (‘phone’ shares a first sound with ‘fence’ and not ‘paper’). It was named the ***Deep Phoneme Composite Score*** and is the mean performance in the Phoneme Judgment Task (C, D) and the deep condition in the Phonemic Manipulation Task.

###### Syllable Number Judgment Task

The Syllable Number Judgment Task also used a picture-based ‘odd-man-out’ paradigm. Participants were asked to select the item whose corresponding English name has a different number of syllables to the other two items. In order to prevent the use of word length as a strategy, words in each triad all contained the same number of letters and were either 5 or 6 letters long. All stimuli were picture-based. The odd man could either have more or fewer syllables than the other two items (e.g., **lemon**/clock/sheep or **glass**/table/paper).

###### Speechreading task

The speechreading task developed by Mohammed et al. (2003, 2006) was adapted to American English by using a native American English speaker to voice the sentences. Participants saw 15 spoken sentences (with no sound). After each sentence, participants had to select one from six pictures that best corresponded to the sentence just viewed. Picture foils were designed such that the observer must comprehend the whole sentence in order to answer correctly. For example, all six pictures that accompanied the sentence ‘They were under the table’ contained tables, three had more than one person, and one had a single person under a table, etc. Three practice sentences were given as preparation.

###### Short-term memory task – serial recall letter span

Separate lists of video stimuli of letters in English and in ASL were presented at a rate of 1 letter/sec. Visual ASL stimuli and audiovisual English stimuli were presented on the computer screen one at a time. ASL stimuli consisted of a native signer fingerspelling a list of letters and English stimuli consisted of a native speaker enunciating a list of letters in English. Lists ranged from 2 to 9 items in length, with two different lists at each length. The letters in the lists were the same as those used in Bavelier et al. (2008). Letters in both English and ASL were selected to be maximally dissimilar within each language in order to avoid phonological similarity effects (i.e., possible English written letters were: M, Y, S, L, R, K, H, G, P; ASL fingerspelled letters were: B, C, D, F, G, K, L, N, S). Participants were asked to repeat back each list in the precise order in which it was presented. The span was defined as the longest list length (L) recalled without mistakes before both list presentations in the next list length (L + 1) contained an error (e.g., if a participant recalled one list at length five correctly, but missed both lists at length six, their span would be five). Serial recall span was measured in each participants’ preferred language (ASL for deaf native signers and English for oral deaf participants).

###### Free recall span

Participants were presented with lists of 16 words in English or in ASL, at the rate of 1 word every 5 s. Stimuli were videos of a native speaker or signer producing the list of 16 words, with a blank screen between each word. After viewing each list, they were required to immediately recall in their preferred language as many words as possible in any order. Each subject saw one list in each language and was told to try their best if it was not in their native language (e.g., spoken English for native signers or ASL for oral deaf). The items in each list were randomly assigned on a subject-by-subject basis from a list of 32 words, in order to avoid unplanned differences in word combinations that would lead one list to being ‘easier’ than the other. The lists used were roughly matched across groups, as much as possible with unequal sample sizes. Here we will only consider performance on the list in each participants’ preferred language (ASL for deaf native signers and English for oral deaf). Measures of span, primacy and recency were derived from this data. Span was defined as the number of items recalled correctly (Rundus and Atkinson, 1970), primacy and recency scores were defined as the number of words recalled from among the first four (primacy) or last four (recency) items of the lists (Murdoch, 1962).

### Results Experiment 2

#### Performance on Individual Tasks

##### Reading comprehension (PIAT grade-equivalent)

There was no main effect of group on reading comprehension scores, *t*(45) = 0.44, *d* = 0.13, *p* = 0.66.

##### Phonological Composite Scores

**FIGURE 5:**
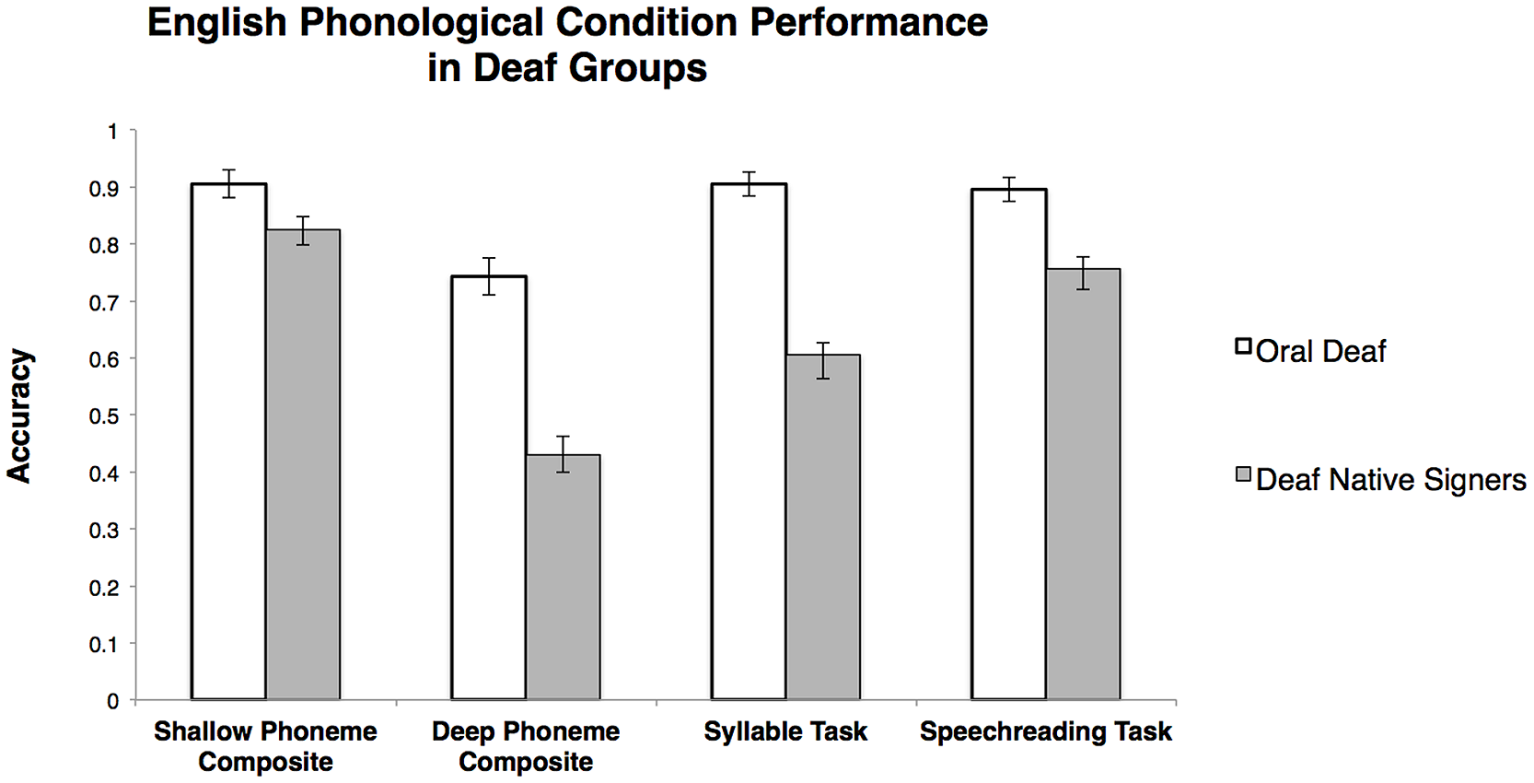
**English Phonological Task Performance.** Error bars represent the SE of the mean.

A 2 × 2 ANOVA on the composite accuracy scores with composite score type (shallow, deep) as a repeated measures and group (deaf native signer, oral deaf) as a between subjects factor revealed, as expected given the previous analyses, main effects of composite score type, *F*(1,45) = 181.83, η^2^ = 0.80, *p* < 0.001, and group, *F*(1,45) = 33.00, η^2^ = 0.42, *p* < 0.001. There was also a significant interaction, *F*(1,45) = 31.43, η^2^ = 0.41, *p* < 0.001,^.^such that the effect of orthographic transparency (deep vs. shallow) was greater for deaf native signers, *t*(25) = 13.70, *d* = 5.48, *p* < 0.001, than it was for the oral deaf, *t*(20) = 5.61, *d* = 2.51, *p* < 0.001 (**Figure 5**).

##### Syllable Number Judgment Task

There was a significant effect of group on accuracy in the Syllable Number Judgment Task, *t*(45) = 5.93, *d* = 1.77, *p* < 0.001, such that the oral deaf group performed significantly better than the deaf native signer group (**Figure 5**).

##### Speechreading Task

There was a significant effect of group on the speechreading task, *t*(45) = 3.09, *d* = 0.92, *p* < 0.001, such that the oral deaf group performed significantly better than the deaf native signer group (**Figure 5**).

##### Serial Recall Memory

**FIGURE 6:**
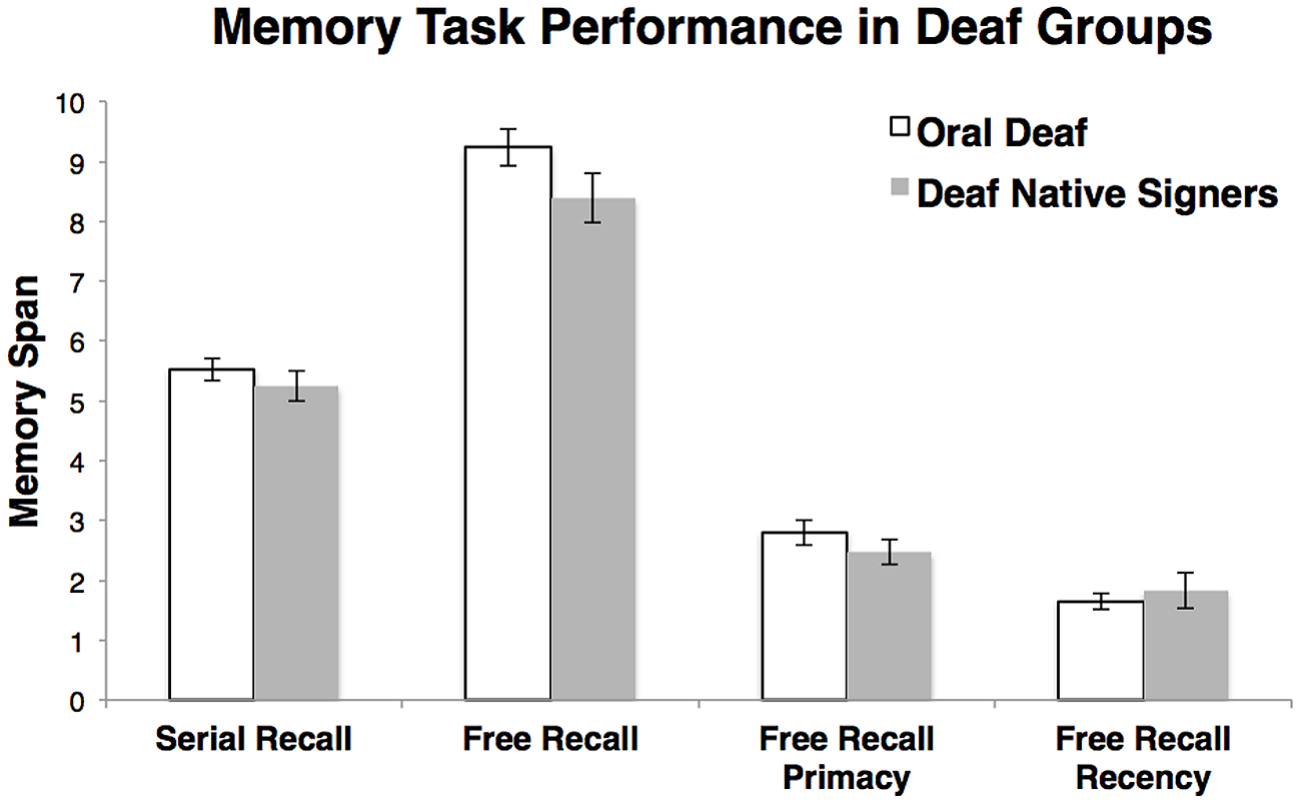
**Memory task performance.** Error bars represent the SE of the mean.

The serial recall spans in deaf native signers and the oral deaf were comparable, *t*(45) = 0.92, *d* = 0.27, *p* = 0.37, and in the range of 5 ± 1 (**Figure 6**), as expected from the existing literature (Boutla et al., 2002, 2004; Koo et al., 2008).

##### Free recall memory

Free recall memory was measured in ASL and in English for each participant. However, here we only include performance in each participant’s preferred language (English for the oral deaf; ASL for deaf native signers). Free recall span was defined as the total number of accurately recalled words from the list. There was no main effect of group, *t*(45) = 1.67, *d* = 0.50, *p* = 0.10. Analyses of the primacy and recency effects also revealed no main effects of group: primacy, *t*(45) = 1.07, *d* = 0.32, *p* = 0.29, and recency, *t*(45) = 0.59, *d* = 0.18, *p* = 0.55 (**Figure 6**)^3^.

A key distinction for our study is that serial recall and primacy free recall tap into different memory processes. Accordingly, these two measures show little correlation in the deaf participants [*r*(45) = 0.143; *p* = 0.34].

#### Predictors of Reading Comprehension

The main question of interest concerns the variables that best predict reading comprehension and whether they differ between the two deaf populations. We first present an analysis of how reading predictors may differ across groups and then consider the impact of the different predictors within each group.

##### Group comparisons

Regression analyses were computed using R (R Development Core Team, 2010) with grade-equivalent PIAT scores as the dependent variable. We first removed all variance in PIAT scores attributable to non-verbal IQ as well as unaided dB loss in both groups, by regressing PIAT scores against TONI-3 scores and the unaided dB loss in the better ear. All further analyses were then performed on the residuals of this regression. Missing data was replaced with the mean, but whether or not missing non-verbal IQ or dB loss data was excluded pairwise or replaced with the mean, the significance levels of the models reported below did not change. Neither non-verbal IQ nor dB loss accounted for a significant amount of variance in any of the models.

**FIGURE 7:**
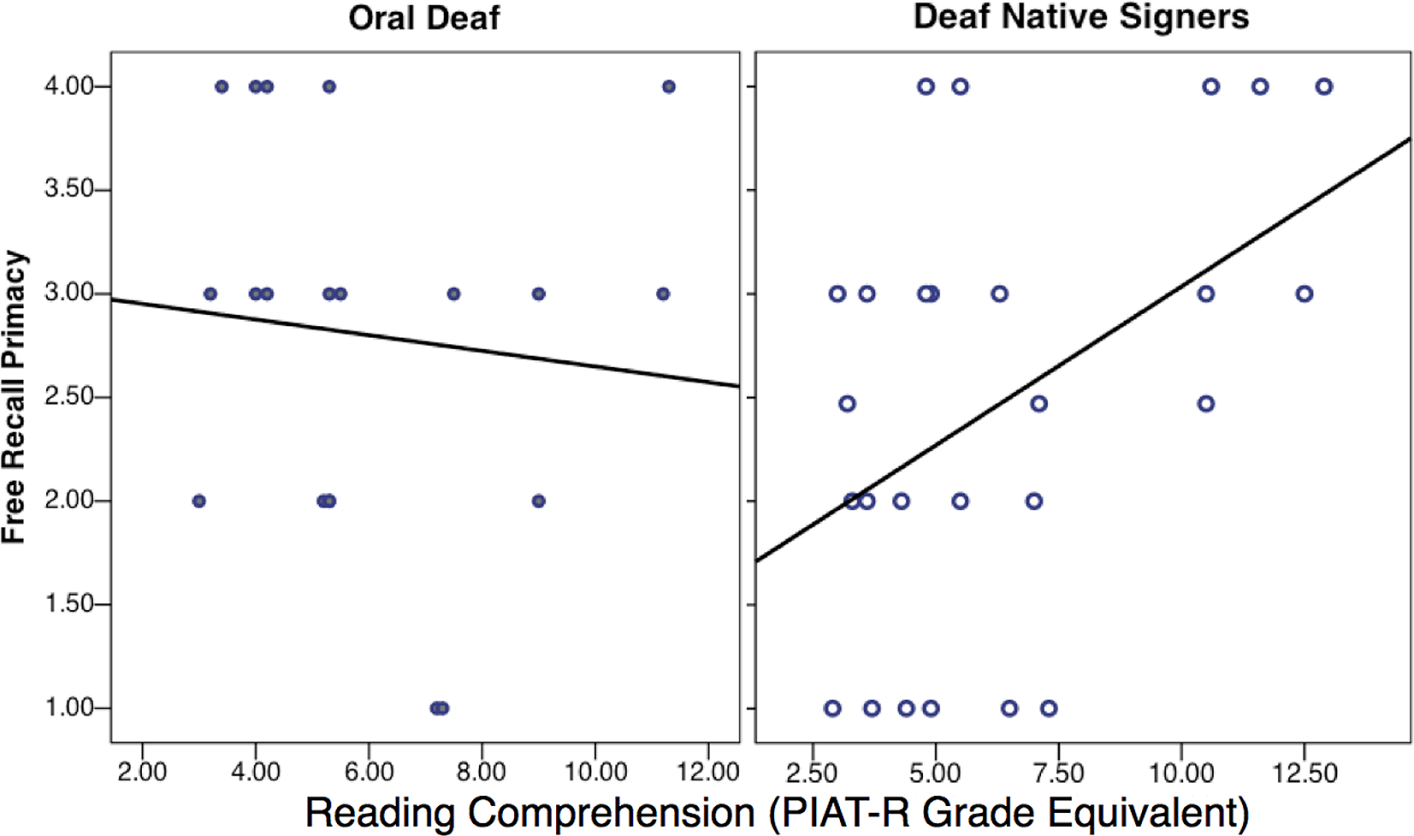
Regression Plots of the Effect of Free Recall Primacy on Reading Comprehension in Deaf Groups.

First, in order to assess whether the predictors of reading comprehension were significantly different across the two deaf groups, two types of regression models were created. Model 1 was a main effect model, with eight predictor variables: Shallow Phoneme Composite Score, Deep Phoneme Composite Score, Syllable Number Judgment Task, Speechreading, Serial Recall, Free Recall Primacy, Free Recall Recency, and group (oral deaf, deaf native signer). Models 2_a-g_ separately added the interaction terms between group and the remaining seven predictors in a stepwise manner. A significant group × predictor interaction term would demonstrate a different level of importance of that given predictor for one group compared to the other. On its own, Model 1 was a significant predictor of reading performance [adjusted *R^2^* = 0.33; *F*(8,36) = 3.67, *p* = 0.003] indicating that together the eight predictors (including group) accounted for a significant amount of variance in reading comprehension across all deaf participants. Interestingly, the group × free recall primacy interaction was the only significant interaction term: *F*(1,35) = 11.59, *p* = 0.002 [Model 2: adjusted *R*^2^ = 0.48; *F*(9,35) = 5.51, *p* < 0.001]. This demonstrates that the free recall primacy measure differentially affects reading comprehension in deaf native signers and oral deaf participants. As can be seen in **Figure 7**, free recall primacy was a better predictor of reading comprehension for deaf native signers than it was for the oral deaf.

There was a significant positive correlation between Free Recall Primacy and Reading Comprehension in the deaf native signers, *R^2^* = 0.21, *p* = 0.02, whereas there was no correlation in the oral deaf, *R^2^* = 0.01, *p* = 0.67. This analysis supports the hypothesis that determinants of reading comprehension are different for oral deaf and deaf native signers. To better understand the main determinants of reading comprehension in each population, each group was considered separately.

##### Individual group partial correlations

To confirm and elaborate on the results of the combined regressions above, partial correlations were separately computed for each group between reading comprehension (having removed variance due to TONI and hearing loss) and the remaining seven predictors: Shallow Phonological Composite Score, Deep Phonological Composite Score, Syllable Number Judgment Task, Speechreading, Serial Recall, Free Recall Primacy, and Free Recall Recency.

The strongest correlations with reading comprehension for the oral deaf were measures of English phonological knowledge, independent of orthographic knowledge. The Deep Phonological score, *r*(18) = 0.66, *p* = 0.003, as well as serial recall span, *r*(18) = 0.50, *p* = 0.04 correlated highly with reading comprehension. None of the other factors were significantly correlated with reading comprehension (all *ps* > 0.12). In stark contrast to the oral deaf, for the deaf native signers the Free Recall Primacy measure, *r*(22) = 0.41, *p* = 0.04, and the Shallow Phonological score, *r*(18) = 0.52, *p* = 0.009, were the only measures that significantly correlated with reading comprehension.

## Discussion

This study compared determinants of reading in two distinct deaf populations with marked differences in language experience. The two deaf groups were selected to differ in their language experience, by recruiting either deaf native signers or oral deaf individuals. Both groups were exposed to a natural language in early childhood, but that language and ongoing language experience was signed in the case of deaf native signers and spoken in the case of the oral deaf. Importantly, these two groups had similar reading comprehension scores, as well as similar performance on general cognitive measures such as non-verbal IQ and free and serial memory recall. However, these two groups differed in what best predicted their reading comprehension scores. Whereas the reading comprehension of the oral deaf was best predicted by both deep phonological knowledge and serial recall span, deaf native signers’ reading comprehension was best predicted by their performance on the free recall task. In particular, reading comprehension in deaf native signers showed a significant correlation with the primacy component of the free recall span, associated with short-term memory consolidation (Bayliss et al., 2015) and semantic coding (Craik and Lockhart, 1972; Martin and Saffran, 1997). More specifically, the link between reading comprehension and the primacy effect in the deaf native signers mirrors that reported by Bauer and Emhert (1984) who found that differences in the primacy effect, compared to the recency effect, better discriminated non-disabled from disabled readers.

### English Phonological Knowledge in Deaf Individuals

There still remains outstanding questions about whether deaf readers, especially oral ones, have qualitatively similar English phonological knowledge to that of hearing individuals. There are different ways that one can acquire English phonological knowledge. It can be acquired from auditory information (such as hearing the difference between a voiced and voiceless glottal stop (/g/ and /k/), from articulatory information as when speaking and speechreading by observing the movement of the lips and mouth, or from a tutored visual experience such as is the case with Cued Speech (LaSasso et al., 2003), or even from orthography during reading in alphabetic language like English. The extant literature on Cued Speech for example makes it clear that such communication training enhances awareness of phonological knowledge for the trained spoken language (Alegria and Lechat, 2005). The resulting phonological knowledge has been shown to be comparable to that of both oral deaf and hearing individuals (Koo et al., 2008) and to facilitate reading skills (Colin et al., 2007). In the present study, our two deaf populations share the fact that they were born profoundly deaf, which makes them different from hearing individuals, but they also differ amongst themselves in their language experience, residual hearing, and use of hearing aids or CIs. Indeed, oral deaf individuals are more likely to attain information from articulation, visual speechreading experience, or aided residual hearing, whereas native signers are most likely acquiring phonological information solely through visual experiences such as reading and limited speechreading. These differences are reflected in the performance of these two groups on the phonological tasks presented in this work. For example, native signers were more likely to perform poorly than the oral deaf in the deep phonological conditions, where orthography was uninformative or misleading.

The current study also provides some insights for cross-linguistic studies of phonological skill in deafness. In addition to the importance of carefully considering population characteristics, we demonstrate that the nature of the orthographic-phonological mapping of a written language may also be important. In light of these considerations, the lack of an effect of language experience (speech versus sign) on phonological awareness in a study conducted in Hebrew is worth considering (Miller, 1997). Hebrew has a relatively simple mapping between orthography and sound and has multiple letters that map onto the same phonemes, like English. Interestingly, conditions that required that type of knowledge (e.g., knowing that when deciding the odd man out between ‘c’, ‘k’ and ‘p’, that ‘c’ and ‘k’ sometimes sound the same) did not reveal major differences between oral and signing deaf participants in the current work. Yet, clearly oral deaf subjects differ from deaf native signers in their knowledge of English phonology. Such differences may not be as easily detectable in a transparent language such as Hebrew.

### Phonological Awareness and Reading Comprehension in Deaf Individuals

The current study also aimed to address concerns about the link between phonological awareness measures and reading scores in two different deaf populations. For the oral deaf, it was the variance in tasks that require English phonological knowledge, above and beyond orthographic knowledge, that best predicted reading. In contrast, for the deaf native signers, in addition to free recall being a good predictor, the measure of phonological skills that best predicted reading was one that could be solved by visual information alone or by conceptual knowledge about spelling. The inclusion of deaf groups with different language experience makes it clear that not all deaf populations possess the same phonological knowledge of English. The use of tasks that systematically manipulated the relationships between phonology and orthography was crucial in being able to draw this conclusion. Our study may explain some of the conflicting reports in the literature (Mayberry et al., 2011) since past studies have included populations that varied significantly in their language experience, all encompassed under the term “deaf.” Furthermore, our study confirms the need to avoid phonological tasks that confound orthographic and phonological knowledge (McQuarrie and Parrila, 2009). The results highlight the importance of a detailed analysis of both the characteristics of the language/script to be read and the population of deaf individuals studied.

The shallow phonological score essentially measures orthographic knowledge or familiarity with spelling, and the usefulness of such information in inferring the phonological structure of English. We did find that it accounted for a significant amount of the variance in reading comprehension in deaf native signers. This score could be linked with single word processing and identification, but without access to more detailed statistics on the participants’ reading habits it is also possible that the shallow phonological score reflects exposure to print, being in a sense an indirect measure of reading skill. Indeed, greater exposure to print could lead to greater orthographic knowledge and better word identification skills, which could in turn lead to overall greater reading skill and comprehension. Further experiments are necessary to clarify the relationship between performance in our shallow phonological conditions, the use of orthography in phonological tasks, and reading comprehension in the deaf.

### Reading Comprehension and Free Recall Memory in Deaf Native Signers

Finally and probably most importantly, the present work indicates that memory processes associated with the free recall task may provide an alternative route for supporting reading in deaf native signers. Primacy scores in the free recall task, associated with semantic processing, was the one predictor that differentially predicted reading comprehension in deaf native signers and the oral deaf. Studies that recruit deaf participants without considering their language experience are likely to encompass only a very small percentage of deaf native signers given their low prevalence, resulting in an over-emphasis on the role of English phonological skills compared to semantic-based memory skill in deaf reading. This may explain why our study is the first one to highlight this link, despite a strong relationship between free recall and reading comprehension in our deaf native signing participants^4^.

These results need to be situated in the larger picture of what we know about reading processes. A first intriguing issue concerns what it may mean for a free recall task tested in American Sign Language to be a good predictor of comprehension of English text in deaf native signers. Due to the connection in the literature between free recall, with a focus on the primacy effect, and semantic processing, one interpretation could be that deaf native signers rely to a greater extent on processing of semantic information at both the word level and the sentence level in the service of reading comprehension. For example, semantic processing is necessary to maintain coherence, hold information online in memory, and make appropriate connections within and between phrase structures in order to comprehend a text. Deficits in semantic processing have been linked to poor comprehension skill (Nation and Snowling, 1998b; Hagtvet, 2003; Cain and Oakhill, 2006). It is possible that enhanced semantic processing, or at least a greater *reliance* on semantic processing (Sinatra et al., 1984; Nation and Snowling, 1998a), may help compensate for deficient phonological skills. Accordingly, top–down semantic influences on deaf readers, such as prior knowledge or context (Kelly, 1995; Jackson et al., 1997) have been shown to be significant predictors of passage comprehension, which is consistent with our current findings. Since ASL grammar is quite different from that of English, deaf native signers not only have to identify words in another language, but they need to understand the syntactic rules that connect them. Yurkowski and Ewoldt (1986) proposed that semantic information maybe crucial in helping with complex syntactic processing.

Another interesting perspective is that deaf native signers are actually bilingual (bi-modal) readers and thus reading their second language when faced with English text (Chamberlain and Mayberry, 2008). Our findings are consistent with the ideas put forth by Ullman (2001, 2005) which suggest that second language learners rely more on lexical memory, supported by the declarative memory system. For example, several studies indicate that non-proficient hearing speakers while reading in their second language differ from first language readers on measures of integration, recognition of aspects of text structure, use of general knowledge, and personal experience, as well as in paying attention to ‘broader phrases’ and keeping the meaning of the passages in mind during reading (Carrell, 1989; Fitzgerald, 1995; Jun Zhang, 2001). Primacy in free recall, also thought to be a measure linked to semantic processing (Craik and Tulving, 1975; Bellezza et al., 1976; Waters and Waters, 1976), could be related to such cognitive skills that highlight the role of recognition and integration of memory representations over broader linguistic units.

## Conclusion

In sum, the present work clarifies the nature of English phonological knowledge in two distinct deaf populations: deaf native signers and the oral deaf. It highlights the importance of considering language experience when evaluating determinants of reading in deaf participants. It also reveals for the first time a potential complementary route to literacy – semantic-based memory – that does not depend upon English phonological skills. It will be for future research to assess precisely how greater reliance on semantic processing may foster good text comprehension, even in the face of poor phonological skills.

## Conflict of Interest Statement

The authors declare that the research was conducted in the absence of any commercial or financial relationships that could be construed as a potential conflict of interest.

## Footnotes

1 One deaf native signer had an unaided hearing loss of 70 dB and one oral deaf had an unaided hearing loss of 63 dB.

2 Two oral deaf became deaf at age 4 years.

3 The stimulus list order was not available for three native signer participants due to a technical malfunction, and their primacy and recency scores were not possible to calculate. Their data was replaced with the group mean for native signers (Primacy = 2.47, Recency = 1.83).

4 In order to ensure that our findings were not a result of a general trend in all deaf readers, regardless of language background, we combined the data from both deaf groups and created high and low median split groups based on their PIAT reading comprehension scores. Both PIAT skill groups were similarly represented by oral deaf and native signers (LowPIAT group contained 11 oral deaf and 12 native signers; HighPIAT group contained 10 oral deaf and 14 native signers). Using these newly defined groups, there were no significant differences in the deep phonological measure (*M*_HighPIAT_ = 0.61, SDHighPIAT = 0.23; MLowPIAT = 0.53, SDLowPIAT = 0.19, *p* = 0.23) or shallow phonological measure (MHighPIAT = 0.89, SDHighPIAT = 0.10; MLowPIAT = 0.83, SDLowPIAT = 0.15, *p* = 0.11). While this may seem surprising given the broad patterns in the literature at large, this result highlights the importance and consequences of combining data from deaf individuals with distinct language backgrounds. A distinctive feature of our study is to have carefully selected groups that have largely homogenous within-group language background and distinct between-group language background. By considering good/bad readers irrespective of language background, we are essentially diluting each of our deaf group’s effects. Accordingly, there were also no significant differences in the free recall primacy scores when considering good/poor readers over the whole deaf group (*M*_HighPIAT_ = 2.69, SDHighPIAT = 1.05; MLowPIAT = 2.54, SDLowPIAT = 0.99, *p* = 0.62). Such results highlight the importance of considering separately the “oral” deaf and the “deaf signers” group.

## References

[B1] Alegria J. Lechat J. (2005). Phonological processing in deaf children: when lipreading and cues are incongruent. *J. Deaf Stud. Deaf Educ.* 10 122–133. 10.1093/deafed/eni013 15778209

[B2] Aparicio M. Gounot D. Demont E. Metz-Lutz M. N. (2007). Phonological processing in relation to reading: an fMRI study in deaf readers. *Neuroimage* 35 1303–1316. 10.1016/j.neuroimage.2006.12.046 17329129

[B3] Baddeley A. D. Lewis V. Vallar G. (1984). Exploring the articulatory loop. *Q. J. Exp. Psychol.* 36A 233–252. 10.1080/14640748408402157

[B4] Bauer R. H. Emhert J. (1984). Information processing in reading-disabled and nondisabled children. *J. Exp. Child Psychol.* 37 271–281. 10.1016/0022-0965(84)90005-5 6726115

[B5] Bavelier D. Newport E. L. Hall M. L. lla T. Boutla M. (2008). Ordered short-term memory differs in signers and speakers: implications for models of short-term memory. *Cognition* 107 433–459. 10.1016/j.cognition.2007.10.012 18083155 PMC2396490

[B6] Bayliss D. M. Bogdanovs J. Jarrold C. (2015). Consolidating working memory: distinguishing the effects of consolidation, rehearsal and attentional refreshing in a working memory span task. *J. Mem. Lang.* 81 34–50. 10.1016/j.jml.2014.12.004

[B7] Bélanger N. N. Baum S. R. Mayberry R. I. (2012a). Reading difficulties in adult deaf readers of French: phonological codes, not guilty! *Sci. Stud. Read.* 16 263–285. 10.1080/10888438.2011.568555

[B8] Bélanger N. N. Slattery T. J. Mayberry R. I. Rayner K. (2012b). Skilled deaf readers have an enhanced perceptual span in reading. *Psychol. Sci.* 23 816–823. 10.1177/0956797611435130 22683830 PMC3723350

[B9] Bélanger N. N. Mayberry R. I. Rayner K. (2013). Orthographic and phonological preview benefits: parafoveal processing in skilled and less-skilled deaf readers. *Q. J. Exp. Psychol.* 66 2237–2252. 10.1080/17470218.2013.780085 PMC3808502 23768045

[B10] Bellezza F. S. Richards D. L. Geiselman R. E. (1976). Semantic processing and organization in free recall. *Mem. Cogn.* 4 415–421. 10.3758/BF03213198 21287383

[B11] Bellugi U. Klima E. Siple P. (1975). Remembering in Signs. *Cognition* 3 93–125. 10.1016/0010-0277(74)90015-8

[B12] Bhatarah P. Ward G. Smith J. Hayes L. (2009). Examining the relationship between free recall and immediate serial recall: similar patterns of rehearsal and similar effects of word length, presentation rate, and articulatory suppression. *Mem. Cogn.* 37 689–713. 10.3758/MC.37.5.689 19487760

[B13] Boutla M. Supalla T. Bavelier D. (2002). What can American Sign Language tell us about capacity limit in working memory? *Paper Presented at the Cognitive Neuroscience Society* San Francisco, CA.

[B14] Boutla M. Supalla T. Newport L. Bavelier D. (2004). Short-term memory span: insights from sign language. *Nat. Neurosci.* 7 997–1002. 10.1038/nn1298 15311279 PMC2945821

[B15] Brown L. (2003). *Test of Nonverbal Intelligence Handbook of Nonverbal Assessment.* (Berlin: Springer) 191–221. 10.1007/978-1-4615-0153-4_10

[B16] Brown L. Sherbenou R. J. Johnsen S. K. (1997). *Test of Nonverbal Intelligence* 3rd Edn Austin, TX: PRO-ED.

[B17] Bruck M. (1992). Persistence of dyslexics’ phonological awareness deficits. *Dev. Psychol.* 28 874 10.1037/0012-1649.28.5.874

[B18] Burgess N. Hitch G. J. (1999). Memory for serial order: a network model of the phonological loop and its timing. *Psychol. Rev.* 106 551 10.1037/0033-295X.106.3.551

[B19] Cain K. Oakhill J. (2006). Profiles of children with specific reading comprehension difficulties. *Br. J. Educ. Psychol.* 76 683–696. 10.1348/000709905X67610 17094880

[B20] Carrell P. L. (1989). Metacognitive awareness and second language reading. *Mod. Lang. J.* 73 121–134. 10.1111/j.1540-4781.1989.tb02534.x

[B21] Chamberlain C. Mayberry R. I. (2000). “Theorizing about the relation between American Sign Language and reading,” in *Language Acquisition by Eye* eds Chamberlain C. Morford J. P. Mayberry R. I. (ahwah, NJ: Earlbaum) 221–259.

[B22] Chamberlain C. Mayberry R. I. (2008). American Sign Language syntactic and narrative comprehension in skilled and less skilled readers: bilingual and bimodal evidence for the linguistic basis of reading. *Appl. Psycholinguist.* 29 367–388. 10.1017/S014271640808017X

[B23] Colin S. Magnan A. Ecalle J. Leybaert J. (2007). Relation between deaf children’s phonological skills in kindergarten and word recognition performance in first grade. *J. Child Psychol. Psychiatry* 48 139–146. 10.1111/j.1469-7610.2006.01700.x 17300552

[B24] Coltheart V. Laxon V. Rickard M. Elton C. (1988). Phonological recoding in reading for meaning by adults and children. *J. Exp. Psychol.* 14 387 10.1037/0278-7393.14.3.387

[B25] Conrad R. (1972). Short-term memory in the Deaf: a test for speech coding. *Br. J. Psychol.* 63 173–180. 10.1111/j.2044-8295.1972.tb02097.x 5045585

[B26] Cornwall A. (1992). The relationship of phonological awareness, rapid naming, and verbal memory to severe reading and spelling disability. *J. Learn. Disabil.* 25 532–538. 10.1177/002221949202500808 1460397

[B27] Craik F. I. M. Lockhart R. S. (1972). Levels of processing: a framework for memory research. *J. Verbal Learning Verbal Behav.* 11 671–684. 10.1016/S0022-5371(72)80001-X

[B28] Craik F. I. M. Tulving E. (1975). Depth of processing and the retention of words in episodic memory. *J. Exp. Psychol.* 104 268–294. 10.1037/0096-3445.104.3.268

[B29] Dallago M. L. L. Moely B. E. (1980). Free recall in boys of normal and poor reading levels as a function of task manipulations. *J. Exp. Child Psychol.* 30 62–78. 10.1016/0022-0965(80)90075-2 7391746

[B30] Dyer A. MacSweeney M. Szczerbinski M. Green L. Campbell R. (2003). Predictors of reading delay in deaf adolescents: the relative contributions of rapid automatized naming speed and phonological awareness and decoding. *J. Deaf Stud. Deaf Educ.* 8 215 10.1093/deafed/eng012 15448050

[B31] Eden G. F. Jones K. M. Cappell K. Gareau L. Wood F. B. Zeffiro T. A. (2004). Neural changes following remediation in adult developmental dyslexia. *Neuron* 44 411–422. 10.1016/j.neuron.2004.10.019 15504323

[B32] Elbro C. Nielsen I. Petersen D. K. (1994). Dyslexia in adults: evidence for deficits in non-word reading and in the phonological representation of lexical items. *Ann. Dyslexia* 44 203–226. 10.1007/BF02648162 24234053

[B33] Erber N. P. (1974). Visual perception of speech by deaf children: recent developments and continuing needs. *J. Speech Hear. Disord.* 39 178–185. 10.1044/jshd.3902.178 4596705

[B34] Figueras B. Edwards L. Langdon D. (2008). Executive function and language in deaf children. *J. Deaf Stud. Deaf Educ.* 13 362–377. 10.1093/deafed/enm067 18252699

[B35] Fitzgerald J. (1995). English-as-a-second-language learners, Äô cognitive reading processes: a review of research in the United States. *Rev. Educ. Res.* 65 145–190. 10.3102/00346543065002145

[B36] Fletcher C. R. (1986). Strategies for the allocation of short-term memory during comprehension. *J. Mem. Lang.* 25 43–58. 10.1016/0749-596X(86)90020-3

[B37] Gabrieli J. D. E. (2009). Dyslexia: a new synergy between education and cognitive neuroscience. *Science* 325 280 10.1126/science.1171999 19608907

[B38] Gallaudet Research Institute. (2005). Available at: https://research.gallaudet.edu/Demographics/

[B39] Gathercole S. E. Willis C. Baddeley A. D. (1991). Differentiating phonological memory and awareness of rhyme: reading and vocabulary development in children. *Br. J. Psychol.* 82 387–406. 10.1111/j.2044-8295.1991.tb02407.x

[B40] Geraci C. Gozzi M. Papagno C. Cecchetto C. (2008). How grammar can cope with limited short-term memory: simultaneity and seriality in sign languages. *Cognition* 106 780–804. 10.1016/j.cognition.2007.04.014 17537417

[B41] Goldin Meadow S. Mayberry R. I. (2001). How do profoundly deaf children learn to read? *Learn. Disabil. Res. Pract.* 16 222–229. 10.1111/0938-8982.00022

[B42] Gozzi M. Geraci C. Cecchetto C. Perugini M. Papagno C. (2011). Looking for an explanation for the low sign span: is order involved? *J. Deaf Stud. Deaf Educ.* 16 101–107. 10.1093/deafed/enq035 20679138

[B43] Hagtvet B. E. (2003). Listening comprehension and reading comprehension in poor decoders: evidence for the importance of syntactic and semantic skills as well as phonological skills. *Read. Writ.* 16 505–539. 10.1023/A:1025521722900

[B44] Hall M. L. Bavelier D. (2010). “Working memory, deafness, and sign language,” in *The Oxford Handbook of Deaf Studies, Language, and Education* Vol. 2 eds Marschark M. Spencer P. E. Nathan P. E. (New York: Oxford University Press) 458–472.

[B45] Hammill D. D. (1987). *Test of Adolescent Language (TOAL-2): A Multidimensional Approach to Assessment: Pro-Ed* Vol. 2 (Austin, TX: Pro-Ed Inc).

[B46] Hanson V. L. Fowler C. A. (1987). Phonological coding in word reading: evidence from hearing and deaf readers. *Mem. Cogn.* 15 199–207. 10.3758/BF03197717 3600259

[B47] Hanson V. L. McGarr N. S. (1989). Rhyme generation by deaf adults. *J. Speech Hear. Res.* 32 2 10.1044/jshr.3201.02 2704195

[B48] Hatcher P. J. Hulme C. Ellis A. W. (1994). Ameliorating early reading failure by integrating the teaching of reading and phonological skills: the phonological linkage hypothesis. *Child Dev.* 65 41–57. 10.2307/1131364

[B49] Hauser P. C. Lukomski J. Hillman T. (2008a). “Development of deaf and hard-of-hearing students’ executive function,” in *Deaf Cognition* eds Marschark M. H. Hauser P. C. (Oxford: Oxford University Press) 286–308.

[B50] Hauser P. C. Paludnevičiene R. Supalla T. Bavelier D. (2008b). “American sign language-sentence reproduction test: development and implications,” in *Sign Language: Spinning and Unraveling the Past, Present and Future* ed. Quadros R. M. D. (Petropolis: Editora Arara Azul) 160–172.

[B51] Hirshorn E. A. Fernandez N. M. Bavelier D. (2012). Routes to short-term memory indexing: lessons from deaf native users of American Sign Language. *Cogn. Neuropsychol.* 29 85–103. 10.1080/02643294.2012.704354 22871205 PMC3472360

[B52] Hogan T. P. Catts H. W. Little T. D. (2005). The relationship between phonological awareness and reading: implications for the assessment of phonological awareness. *Lang. Speech Hear. Serv. Sch.* 36 285–293. 10.1044/0161-1461(2005/029) 16389701 PMC2848754

[B53] Høien T. Lundberg I. Stanovich K. E. Bjaalid I.-K. (1995). Components of phonological awareness. *Read. Writ.* 7 171–188. 10.1007/BF01027184

[B54] Hyde T. S. Jenkins J. J. (1973). Recall for words as a function of semantic, graphic, and syntactic orienting tasks1. *J. Verbal Learning Verbal Behav.* 12 471–480. 10.1016/S0022-5371(73)80027-1

[B55] Jackson D. Paul P. Smith J. (1997). Prior knowledge and reading comprehension ability of deaf adolescents. *J. Deaf Stud. Deaf Educ.* 2 172 10.1093/oxfordjournals.deafed.a014323 15579846

[B56] Jolicœur P. Dell’Acqua R. (1998). The demonstration of short-term consolidation. *Cogn. Psychol.* 36 138–202. 10.1006/cogp.1998.0684 9721199

[B57] Jun Zhang L. (2001). Awareness in reading: EFL students’ metacognitive knowledge of reading strategies in an acquisition-poor environment. *Lang. Awareness* 10 268–288. 10.1080/09658410108667039

[B58] Keenan J. M. Betjemann R. S. Wadsworth S. J. DeFries J. C. Olson R. K. (2006). Genetic and environmental influences on reading and listening comprehension. *J. Res. Read.* 29 75–91. 10.1111/j.1467-9817.2006.00293.x

[B59] Kelly L. P. (1995). Processing of bottom-up and top-down information by skilled and average deaf readers and implications for whole language instruction. *Except. Child.* 61 318–334.

[B60] Koo D. Crain K. LaSasso C. Eden G. F. (2008). Phonological awareness and short term memory in hearing and deaf individuals of different communication backgrounds. *Ann. N. Y. Acad Sci.* 1145 83–99. 10.1196/annals.1416.025 19076391

[B61] Landi N. (2010). An examination of the relationship between reading comprehension, higher-level and lower-level reading sub-skills in adults. *Read. Writ.* 3 701–717. 10.1007/s11145-009-9180-z 21691452 PMC3117585

[B62] LaSasso C. Crain K. Leybaert J. (2003). Rhyme generation in deaf students: the effect of exposure to cued speech. *J. Deaf Stud. Deaf Educ.* 8 250–270. 10.1093/deafed/eng014 15448052

[B63] Lee J. F. (1986). On the use of the recall task to measure L2 reading comprehension. *Stud. Second Lang. Acquis.* 8 201–212. 10.1017/S0272263100006082

[B64] Lichtenstein E. H. (1998). The relationships between reading processes and English skills of deaf college students. *J. Deaf Stud. Deaf Educ.* 3 80 10.1093/oxfordjournals.deafed.a014348 15579859

[B65] Luetke-Stahlman B. Nielsen D. C. (2003). The contribution of phonological awareness and receptive and expressive English to the reading ability of deaf students with varying degrees of exposure to accurate English. *J. Deaf Stud. Deaf Educ.* 8 464 10.1093/deafed/eng028 15448076

[B66] MacSweeney M. Brammer M. J. Waters D. Goswami U. (2009). Enhanced activation of the left inferior frontal gyrus in deaf and dyslexic adults during rhyming. *Brain* 132 1928–1940. 10.1093/brain/awp129 19467990 PMC2702837

[B67] MacSweeney M. Waters D. Brammer M. J. Woll B. Goswami U. (2008). Phonological processing in deaf signers and the impact of age of first language acquisition. *Neuroimage* 40 1369–1379. 10.1016/j.neuroimage.2007.12.047 18282770 PMC2278232

[B68] Markwardt F. C. (1989). *Peabody Individual Achievement Test-Revised: PIAT-R.* Circle Pines, MN: American Guidance Service.

[B69] Marshall C. R. Mann W. Morgan G. (2011). Short-term memory in signed languages: not just a disadvantage for serial recall. *Front. Psychol.* 2:102 10.3389/fpsyg.2011.00102 PMC3132676 21779259

[B70] Martin N. Gupta P. (2004). Exploring the relationship between word processing and verbal short-term memory: evidence from associations and dissociations. *Cogn. Neuropsychol.* 21 213–228. 10.1080/02643290342000447 21038201

[B71] Martin N. Saffran E. M. (1997). Language and auditory-verbal short-term memory impairments: evidence for common underlying processes. *Cogn. Neuropsychol.* 14 641–682. 10.1080/026432997381402

[B72] Mayberry R. I. (1993). First-language acquisition after childhood differs from second-language acquisition: the case of American Sign Language. *J. Speech Lang. Hear. Res.* 36 1258 10.1044/jshr.3606.1258 8114493

[B73] Mayberry R. I. del Giudice A. A. Lieberman A. M. (2011). Reading achievement in relation to phonological coding and awareness in deaf readers: a meta-analysis. *J. Deaf Stud. Deaf Educ.* 16 164–188. 10.1093/deafed/enq049 21071623 PMC3739043

[B74] McCardle P. Scarborough H. S. Catts H. W. (2001). Predicting, explaining, and preventing children’s reading difficulties. *Learn. Disabil. Res. Pract.* 16 230–239. 10.1111/0938-8982.00023

[B75] McDougall S. Hulme C. Ellis A. Monk A. (1994). Learning to read: the role of short-term memory and phonological skills. *J. Exp. Child Psychol.* 58 112–133. 10.1006/jecp.1994.1028 8064216

[B76] McQuarrie L. Parrila R. (2009). Phonological representations in deaf children: rethinking the functional equivalence hypothesis. *J. Deaf Stud. Deaf Educ.* 14 137 10.1093/deafed/enn025 18635579

[B77] Melby-Lervåg M. Hulme C. (2010). Serial and free recall in children can be improved by training evidence for the importance of phonological and semantic representations in immediate memory tasks. *Psychol. Sci.* 21 1694–1700. 10.1177/0956797610385355 20921571

[B78] Miller P. (1997). The effect of communication mode on the development of phonemic awareness in prelingually deaf students. *J. Speech Lang. Hear. Res.* 40 1151 10.1044/jslhr.4005.1151 9328886

[B79] Miller P. Clark M. D. (2011). Phonemic awareness is not necessary to become a skilled deaf reader. *J. Dev. Phys. Disabil.* 23 459–476. 10.1007/s10882-011-9246-0

[B80] Mohammed T. Campbell R. Macsweeney M. Barry F. Coleman M. (2006). Speechreading and its association with reading among deaf, hearing and dyslexic individuals. *Clin. Linguist. Phon.* 20 621–630. 10.1080/02699200500266745 17056494

[B81] Mohammed T. E. MacSweeney M. Campbell R. (2003). “Developing the TAS: individual differences in silent speechreading, reading and phonological awareness in deaf and hearing speechreaders,” in *Proceedings of the AVSP 2003 – International Conference on Audio-Visual Speech Processing* Saint-Jorioz 49–54.

[B82] Morere D. A. (2012). “Measures of reading achievement,” in *Assessing Literacy in Deaf Individuals* eds Allen T. Morere D. A. (New York: Springer) 107–126. 10.1007/978-1-4614-5269-0_6

[B83] Morford J. P. Wilkinson E. Villwock A. Piñar P. Kroll J. F. (2011). When deaf signers read English: do written words activate their sign translations? *Cognition* 118 286–292. 10.1016/j.cognition.2010.11.006 21145047 PMC3034361

[B84] Murdoch B. B. (1962). The serial position effect of free recall. *J. Exp. Psychol.* 64 482–488. 10.1037/h0045106

[B85] Nation K. Snowling M. J. (1998a). Individual differences in contextual facilitation: evidence from dyslexia and poor reading comprehension. *Child Dev.* 69 996–1011. 10.1111/j.1467-8624.1998.tb06157.x 9768483

[B86] Nation K. Snowling M. J. (1998b). Semantic processing and the development of word-recognition skills: evidence from children with reading comprehension difficulties. *J. Mem. Lang.* 39 85–101. 10.1006/jmla.1998.2564

[B87] Padden C. Ramsey C. (2000). “American sign language and reading ability in deaf children,” in *Language Acquisition by Eye* eds Chamerlain C. Morford J. P. Mayberry R. I. (Mahwah, NJ: Lawrence Erlbaum Associates Publishers) 165–189.

[B88] Parrila R. Kirby J. R. McQuarrie L. (2004). Articulation rate, naming speed, verbal short-term memory, and phonological awareness: longitudinal predictors of early reading development? *Sci. Stud. Read.* 8 3–26. 10.1207/s1532799xssr0801_2

[B89] Pennington B. F. Bishop D. V. (2009). Relations among speech, language, and reading disorders. *Annu. Rev. Psychol.* 60 283–306. 10.1146/annurev.psych.60.110707.163548 18652545

[B90] Perfetti C. A. Hart L. (2001). “The lexical quality hypothesis,” in *Precursors of Functional Literacy* Vol. 11 eds Elbro C. Reitsma P. Verhoeven L. (Amsterdam: John Benjamins) 67–86.

[B91] Perfetti C. A. Landi N. Oakhill J. (2005). “The acquisition of reading comprehension skill,” in *The Science of Reading: A Handbook* eds Snowling M. J. Hulme C. (Oxford: Blackwell Publishing Ltd). 227–247.

[B92] Piñar P. Dussias P. E. Morford J. P. (2011). Deaf readers as bilinguals: an examination of deaf readers’ print comprehension in light of current advances in bilingualism and second language processing. *Lang. Linguist. Compass* 5 691–704. 10.1111/j.1749-818X.2011.00307.x

[B93] Prinz P. M. Strong M. (1998). ASL proficiency and English literacy within a bilingual deaf education model of instruction. *Top. Lang. Disord.* 18 47 10.1097/00011363-199808000-00006

[B94] R Development Core Team. (2010). *R: A Language and Environment for Statistical Computing.* Vienna: R Foundation for Statistical Computing.

[B95] Rundus D. Atkinson R. C. (1970). Rehearsal processes in free recall: a procedure for direct observation. *J. Verbal Learn. Verbal Behav.* 9 99–105. 10.1016/S0022-5371(70)80015-9

[B96] Scarborough H. S. (2009). “Connecting early language and literacy to later reading (dis) abilities: evidence, theory, and practice,” in *Approaching Difficulties in Literacy Development: Assessment, Pedagogy, and Programmes* eds Fletcher-Campbell F. Soler J. Reid G. (Milton Keynes: Sage) 23–39.

[B97] Shankweiler D. Liberman I. Y. (eds). (1989). “How problems of comprehension are related to difficulties in decoding,” in *Phonology and Reading Disability: Solving the reading Puzzle* (Ann Arbor: University of Michigan Press) 35–67 .

[B98] Shaywitz B. A. Shaywitz S. E. Blachman B. A. Pugh K. R. Fulbright R. K. Skudlarski P. (2004). Development of left occipitotemporal systems for skilled reading in children after a phonologically-based intervention. *Biol. Psychiatry* 55 926–933. 10.1016/j.biopsych.2003.12.019 15110736

[B99] Siegel L. S. Linder B. A. (1984). Short-term memory processes in children with reading and arithmetic learning disabilities. *Dev. Psychol.* 20 200–207. 10.1037/0012-1649.20.2.200

[B100] Sinatra R. C. Stahl-Gemake J. Berg D. N. (1984). Improving reading comprehension of disabled readers through semantic mapping. *Read. Teach.* 38 22–29.

[B101] Snowling M. (1998). Dyslexia as a phonological deficit: Evidence and implications. *Child Adoles. Ment. Health* 3 4–11. 10.1111/1475-3588.00201

[B102] Stahl S. A. Murray B. A. (1994). Defining phonological awareness and its relationship to early reading. *J. Educ. Psychol.* 86 221–234. 10.1037/0022-0663.86.2.221

[B103] Sterne A. Goswami U. (2000). Phonological awareness of syllables, rhymes, and phonemes in deaf children. *J. Child Psychol. Psychiatry Allied Discipl.* 41 609–625. 10.1111/1469-7610.00648 10946753

[B104] Supalla T. Hauser P. C. Bavelier D. (2014). Reproducing American Sign Language sentences: cognitive scaffolding in working memory. *Front. Psychol.* 5:859 10.3389/fpsyg.2014.00859 PMC4126373 25152744

[B105] Swanson H. L. (1999). Reading comprehension and working memory in learning-disabled readers: is the phonological loop more important than the executive system? *J. Exp. Child Psychol.* 72 1–31. 10.1006/jecp.1998.2477 9888984

[B106] Swanson H. L. Ashbaker M. H. (2000). Working memory, short-term memory, speech rate, word recognition and reading comprehension in learning disabled readers: does the executive system have a role? *Intelligence* 28 1–30. 10.1016/S0160-2896(99)00025-2

[B107] Swanson H. L. Howell M. (2001). Working memory, short-term memory, and speech rate as predictors of children’s reading performance at different ages. *J. Educ. Psychol.* 93 720 10.1037/0022-0663.93.4.720

[B108] Traxler C. B. (2000). The stanford achievement test 9th edition: national norming and performance standards for deaf and hard of hearing students. *J. Deaf Stud. Deaf Educ.* 5 337–348. 10.1093/deafed/5.4.337 15454499

[B109] Trezek B. J. Wang Y. Paul P. V. (2010). *Reading and Deafness: Theory, Research, and Practice.* Clifton Park, NY: Cengage Learning.

[B110] Ullman M. T. (2001). The neural basis of lexicon and grammar in first and second language: the declarative procedural model. *Bilingualism* 4 105–122. 10.1017/S1366728901000220

[B111] Ullman M. T. (2005). “A cognitive neuroscience perspective on second language acquisition: the declarative/procedural model,” in *Mind and Context in Adult Second Language Acquisition: Methods, Theory, and Practice* ed. Sanz C. (Washington, DC: Georgetown University Press) 141–178.

[B112] Wagner R. K. Torgesen J. K. (1987). The nature of phonological processing and its causal role in the acquisition of reading skills. *Psychol. Bull.* 101 192–212. 10.1037/0033-2909.101.2.192

[B113] Wagner R. K. Torgesen J. K. Rashotte C. A. (1994). Development of reading-related phonological processing abilities: new evidence of bidirectional causality from a latent variable longitudinal study. *Dev. Psychol.* 30 73–87. 10.1037/0012-1649.30.1.73

[B114] Wagner R. K. Torgesen J. K. Rashotte C. A. Hecht S. A. Barker T. A. Burgess S. R. (1997). Changing relations between phonological processing abilities and word-level reading as children develop from beginning to skilled readers: a 5-year longitudinal study. *Dev. Psychol.* 33 468–478. 10.1037/0012-1649.33.3.468 9149925

[B115] Walden B. E. Grant K. W. Cord M. T. (2001). Effects of amplification and speechreading on consonant recognition by persons with impaired hearing. *Ear Hear.* 22 333–341. 10.1097/00003446-200108000-00007 11527039

[B116] Waters H. S. Waters E. (1976). Semantic processing in children’s free recall: evidence for the importance of attentional factors and encoding variability. *J. Exp. Psychol.* 2 370–380. 10.1037/0278-7393.2.4.370

[B117] Yurkowski P. Ewoldt C. (1986). A case for the semantic processing of the deaf reader. *Am. Ann. Deaf* 131 243–247. 10.1353/aad.2012.0795 3751826

