# Supplementary material for: The contribution of phonological knowledge, memory, and language background to reading comprehension in deaf populations
Source: Front Psychol. 2015 Aug 25;6:1153. doi: 10.3389/fpsyg.2015.01153 (PMC4548088; doi:10.3389/fpsyg.2015.01153)
Supplement: Supplementary file 1 [file Table_1.DOCX]

Table 1. Demographic and language backgrounds of participants (mean scores with ranges or standard deviations).
