# Supplementary material for: The contribution of phonological knowledge, memory, and language background to reading comprehension in deaf populations
Source: Front Psychol. 2015 Aug 25;6:1153. doi: 10.3389/fpsyg.2015.01153 (PMC4548088; doi:10.3389/fpsyg.2015.01153)
Supplement: Supplementary file 2 [file Data_Sheet_1.DOCX]

**Supplemental Information**

**Hearing Participants**

Twenty-one hearing participants (*M*_age_ = 24 (18-33); 10 female) were enrolled to validate the phonological tasks in Experiment 1A. They were recruited to have no more than a high-school diploma in an effort to match their reading skill with the deaf groups under study. We note that reading comprehension as measured by the PIAT-R test, remained, however, slightly better in the hearing (mean PIAT Grade Equivalent = 8.1 ± 2.8). Most hearing participants were employed and/or taking some college classes.

**Phoneme Judgment Task**

A 4 x 2 ANOVA was conducted with *orthographic transparency* (A, B, C, D) and *phoneme type* (consonant, vowel) as repeated measures on data collected from the hearing participants (Figure S1). The main effect of orthographic transparency, *F*(3,60)= 7.40, *η^2^*=.27, *p*<.001, was significant in the predicted direction: the conditions that could be solved by transparent orthography alone were more accurate than those that required knowledge of orthographic-to-phonological regularities, with the condition where an orthographic strategy would lead to consistently incorrect responses being the worst. None of the other effects were significant.

**
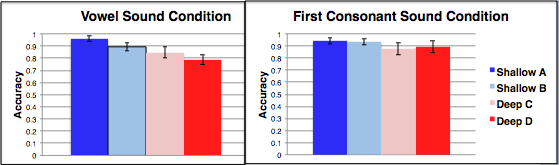
Figure S1.** Hearing Participants’ Performance on Phoneme Judgment Task. Error bars represent standard error of the mean.

**Phonemic Manipulation Task**

Data from the Phonemic Manipulation Task were entered into a paired t-test comparing the two levels of orthographic transparency (shallow, deep) in the hearing participants. There was a significant main effect of orthographic transparency, *t*(20)= 2.59, *d*=1.16 , *p*=. 018, such that participants were less accurate for the deep condition (where a transparent orthographic strategy could not be used successfully) compared to the shallow condition (where a transparent orthographic strategy could be used) (Figure S2).

**Figure S2.** Hearing Participants’ Performance on Phonemic Manipulation Task. Error bars represent standard error of the mean.
